# Role of phospho–ezrin in differentiating thyroid carcinoma

**DOI:** 10.1038/s41598-019-42612-0

**Published:** 2019-04-17

**Authors:** Lakshmi Mohan Lathika, Jagathnath Krishna Kumarapillai Mohanan Nair, Valliamma Neelakandapilla Saritha, Kunjuraman Sujathan, Sreeharshan Sreeja

**Affiliations:** 1 Cancer Research Program, Rajiv Gandhi Centre for Biotechnology, Thiruvananthapuram, Kerala India; 2 Cancer Epidemiology & Biostatistics, Regional Cancer Centre, Thiruvananthapuram, Kerala India; 3 Cancer Research, Regional Cancer Centre, Thiruvananthapuram, Kerala India

**Keywords:** Thyroid cancer, Head and neck cancer, Metastasis

## Abstract

Comprehensive theory explaining the relationship between estrogen (E2) and ezrin in metastasis of thyroid cancer remains non-elicited. *In vitro* results revealed that E2 could stimulate the expression and phosphorylation of ezrin in a time and dose dependent manner. Our data clearly showed that E2 enhanced the migration and invasion of cells, which was reversed by the transfection of cells with ezrin specific siRNA. Further, we observed that Phosphoinositide 3-kinase (PI3K) ROCK-2 are among the kinases responsible for E2 induced phosphorylation of ezrin. Clinical validation of ezrin/phospho-ezrin revealed that phospho-ezrin was intensely expressed in follicular thyroid carcinoma (FTC) and follicular variant of papillary thyroid carcinoma (FVPTC), while it was completely absent in follicular adenoma (FA) lesions in which the differentiation of the follicular neoplasms remains subtle. When histology of different carcinomas is correlated with benign FA with respect to phospho-ezrin, we observed that the marker was highly significant (p = 0.0001). 100% sensitivity, specificity and diagnostic accuracy of the above marker in the histological association of FTC, FVPTC with FA, enables us to suggest phospho-ezrin as a diagnostic marker to differentiate the follicular neoplasms. These data are the first to suggest the dynamic regulation of ezrin phosphorylation during metastasis in FTC.

## Introduction

Thyroid carcinoma is 3–4 times more prevalent in women than in men^1^. The role of sex hormone estrogen (E2) in this gender discrepancy and in the malignancy of thyroid carcinoma is well established^2,3^. It is clearly demonstrated that like breast and ovary, thyroid is also an estrogen responsive tissue^4,5^. In fact, the growth promoting effect of estrogen was observed in the thyroid cells derived from both males and females which accentuate the importance of hormone rather than any other sex factors^6^. Cancer cell metastasis and invasion is characterized by cell migration and dissemination to stroma through the spontaneous formation of structures like membrane ruffles, filopodia and lamellipodia^7^. These events depend on rapid activation of ezrin, radixin, moesin (ERM) family of proteins. Ezrin belongs to Ezrin, Radixin and moesin (ERM) family of proteins of band 4.1 superfamily that crosslinks the actin cytoskeleton which forms the basis of those structures^8^. Despite other ERM proteins, ezrin knock down in osteosarcoma cells of both human and mouse models resulted in inhibition of metastasis^9,10^. This indicates that ezrin has an exclusive role in cells undergoing metastasis. Ezrin is overexpressed in metastatic murine rhabdomyosarcoma and osteosarcoma cell lines when compared to poorly metastatic counterparts^11^. Functional activation of ERM proteins occurs by phosphorylation of Threonin at C-terminal residue; T567 in ezrin, T564 in radixin and T558 in moesin^12^. This mode of activation plays a central role in cytoskeletal remodelling by collaborating with actin filaments and regulate diversified processes like adhesion, cell motility, survival and morphogenesis^13^. Several protein kinases like protein kinase C (PKC), G- protein coupled receptor kinase -2 (GRK-2) are responsible for the activation of ezrin^14^.

Recently it is identified that estrogens have significant impact on regulating the cytoskeletal components and subsequent interaction with extracellular environment, cell movement etc in estrogen responsive tissues^15,16^. Aberrant expression of ezrin turns on the estrogen sensitive breast and ovarian metastasis suggesting that ezrin is a definitive target of action^17^. In this study, we found that phosphorylated ezrin is significantly activated by estrogen and more expression was found in invading areas of follicular thyroid carcinoma. However, studies regarding the extrathyroidal invasion of thyroid carcinoma are in the most trivial stage. For disclosing the reason for the extrathyroidal invasion, *in vitro* experiments were performed in normal thyroid (Nthy-ori3-1) cells as well as in follicular thyroid cancer (FTC-133) cells and observed that estrogen has a key role in inducing the expression of ezrin in both the cell lines. In this study, we delineated the estrogen induced phospho-regulating pathway of ezrin via Rho associated kinase-2 (ROCK -2). We carried out immunohistochemical analysis of ezrin and phospho-ezrin (T567) in patient samples of papillary thyroid carcinoma (PTC), follicular thyroid carcinoma (FTC), follicular variant of papillary thyroid carcinoma (FVPTC) and follicular adenoma (FA). We observed an overexpression of phospho-ezrin in FTC and FVPTC while, the marker was completely absent in FA. This study has thrown light into the dilemma of differential diagnosis of follicular neoplasms in which, the cytologic features overlap among these lesions and the differential diagnosis is problematic for clinicians. We observed 100% sensitivity, specificity and diagnostic accuracy for the marker phospho-ezrin in the histological correlation of FTC and FVPTC with FA which enables us to suggest phospho-ezrin as a diagnostic marker to differentially diagnose the follicular neoplasms.

## Results

### Expression of Estrogen Receptor alpha (ERα) in thyroid cells

**Figure 1:**
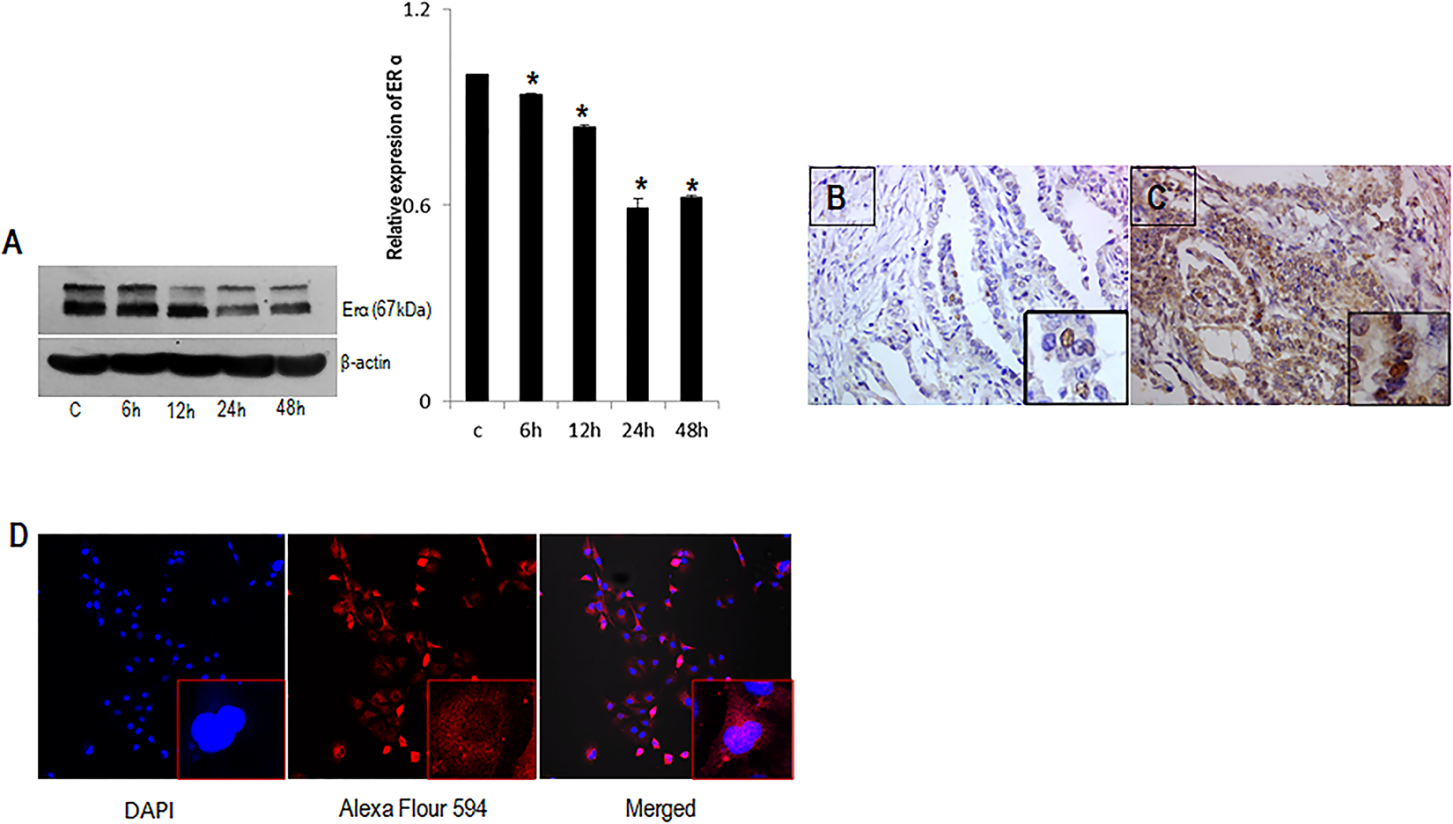
Presence of ERα in FTC-133 cells and thyroid lesions. (**A**) FTC-133 cells were exposed to E2 (10 nM) for 6, 12, 24 and 48 hours respectively. Representative image of western blot showing immunoreactive protein ERα in FTC-133 cells. Representative micrographs of immunohistochemical expression of ERα in (**B**) papillary thyroid carcinoma (**C**) and its follicular variant lesions. (**D**) Immunolocalization of ERα in FTC-133 cells. Images showing DAPI nuclear staining, Alexa flour 594 staining and merged. Images were analysed by NIS software. The western blot shown here is representative of three independent experiments with similar results. β-actin is selected as the loading control. ERα densitometry values were normalized to the values of β- actin. Statistical significance was calculated using t-test *p < 0.05; vs. corresponding control.

So far, the expression of ERα (Estrogen receptor α) in follicular thyroid cancer cells is sparsely reported. Since, it is documented that effects of E2 are fundamentally regulated by direct binding to the estrogen receptors (ERs), and ERβ (Estrogen receptor β) is negatively correlated with proliferation in thyroid cells^18^, we investigated the presence of ERα in FTC cells after treatment with E2 (10 nM) until 48 hours. We observed a gradually decreasing pattern of ERα in FTC cells which may be due to the turnover of the protein after E2 treatment (Fig. 1A). Further, the localization of ERα in FTC cells were confirmed by immunocytochemistry. Confocal microscopy revealed that ERα was mainly localized in perinuclear cytoplasmic ring and the cytoplasm of the cells (Fig. 1D). Moreover, the expression of ERα in PTC and Nthy cells was documented from our lab previously^19^. On clinical validation of presence of ERαin tissue samples obtained from different thyroid lesions, expression of ERα was confined to only three samples of PTC (Fig. 1B) and its follicular variants (Fig. 1C) alone, demonstrating prominent nuclear and cytoplasmic staining. None of the FTC lesions were positive for ERα.

### E2 enhanced the expression of ezrin via ERα 

**Figure 2:**
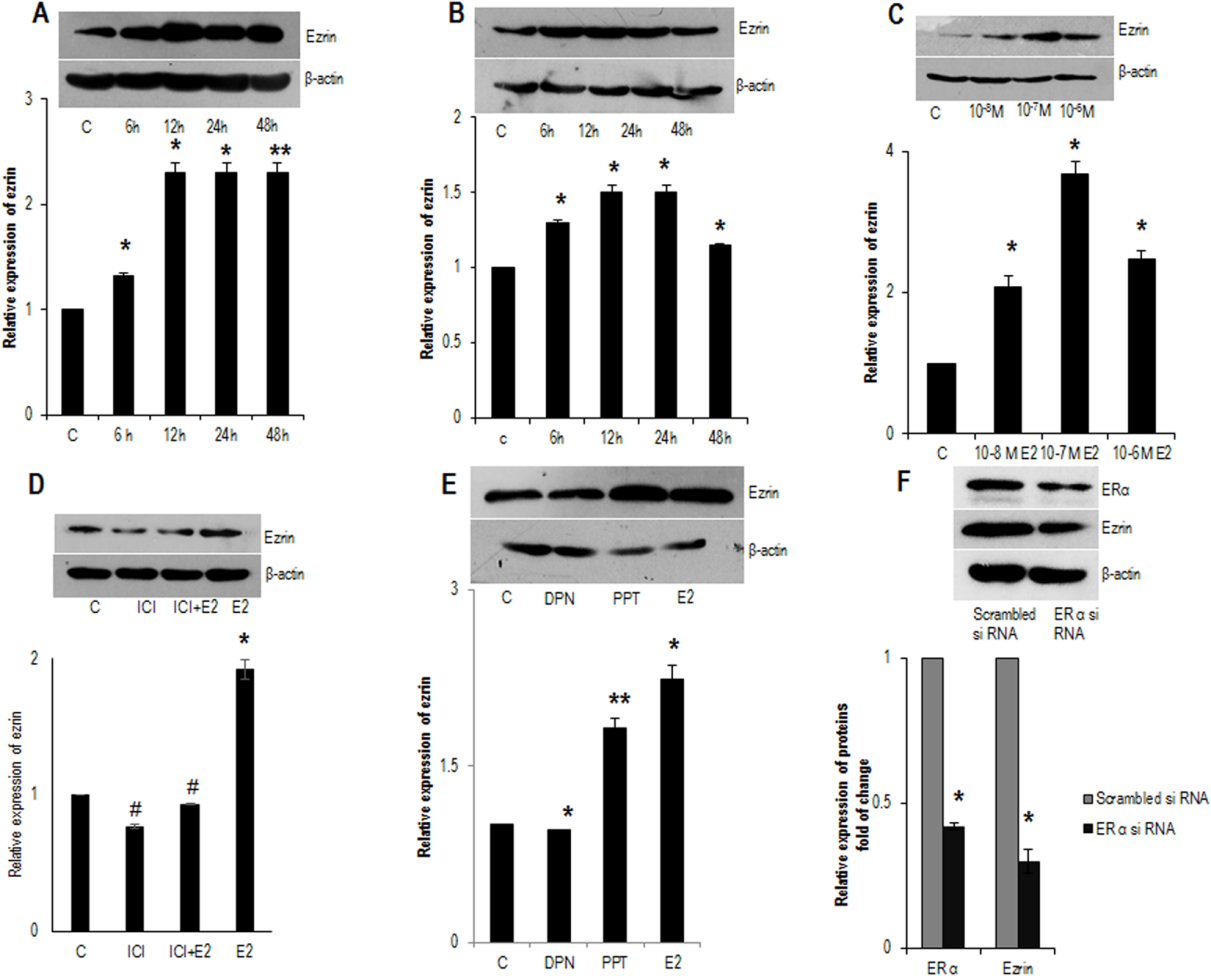
Time and dose response of ezrin to E2 treatment. Western blot analysis showing expression of ezrin in (**A**) FTC-133 and (**B**) Nthy cells in response to E2 (10 nM) treatment until 48 hours. (**C**) Western blot analysis showing expression of ezrin with increasing concentrations of E2 (10^−8^ M, 10^−7^ M, and 10^−6^ M). (**D**) Western blot showing the expression of ezrin in FTC-133 cells treated with anti-estrogen ICI 182780 in presence or absence of E2 (10 nM) for 24 hours. (**E**) FTC-133 cells exposed to E2 (10 nM), ER β agonist DPN (10 nM) and ER α agonist PPT (10 nM). 24 hours post treatment, lysates were taken and analysed by western blot. (**F**) Western blot showing expression of ezrin in ERα silenced FTC cells. β-actin is selected as the loading control. Ezrin densitometry values were normalized to the values of β- actin. The western blots shown here are representative of three independent experiments with similar results. Statistical significance was calculated using t-test *p < 0.05; **p < 0.01 vs. corresponding control; ^#^p < 0.05, ^##^p < 0.05 vs. E2 respectively.

The regulatory role of E2 on ezrin is established in several estrogen-responsive tissues like breast and ovary^20,21^. To study the effect of E2 on ezrin, ERα positive FTC-133 and normal thyroid cells (Nthy) were treated with 10 nM E2 for different time intervals 6 h, 12 h, 24 h, 48 h respectively. Quite interestingly, it was found that ezrin expression was induced after the treatment with E2 in both FTC-133 (Fig. 2A) and Nthy (Fig. 2B) cells compared to the untreated control. The induction was more persistent and significant in FTC cells compared to the induction in Nthy cells (Fig. 2A). Concurrently, expression of ezrin was found to be increasing in FTC-133 cells with increasing doses of E2 for 24 hours (Fig. 2C).

As thyroid is an estrogen responsive tissue, it expresses the ER subtypes ERα and ERβ. To confirm estrogen receptors are involved in induction of ezrin via ERα, we treated FTC-133 cells with E2 (10 nM) and an anti-estrogen ICI-182780 (1 µM) in presence or absence of E2 (10 nM). Treatment with ICI-182780 significantly reduced the expression of ezrin, suggesting that estrogenic action occurs via ERs (Fig. 2D). To identify the ER subtype more active in E2 induced expression of ezrin, FTC-133 cells were exposed to non-subtype selective agonist E2 (10 nM), and ERα subtype selective ligand propyl pyrazole trizol (PPT) (10 nM) and ERβ subtype selective ligand diarylpropionitrile (DPN) (10 nM). The treatment with PPT resulted in robust, stimulation of ezrin in FTC cells with a similar efficacy of E2 (Fig. 2E) suggesting that ERα has the key role in the stimulation. The ERβ agonist DPN did not cause any stimulation of ezrin and this data supports the fact that ERβ having suppressive role in extrathyroidal extension^18^. To confirm this, we investigated the expression of ezrin in ERα silenced FTC cells and we observed ERα suppression attenuated the expression of ezrin confirming the role of ERα in ezrin (Fig. 2F).

**Figure 3:**
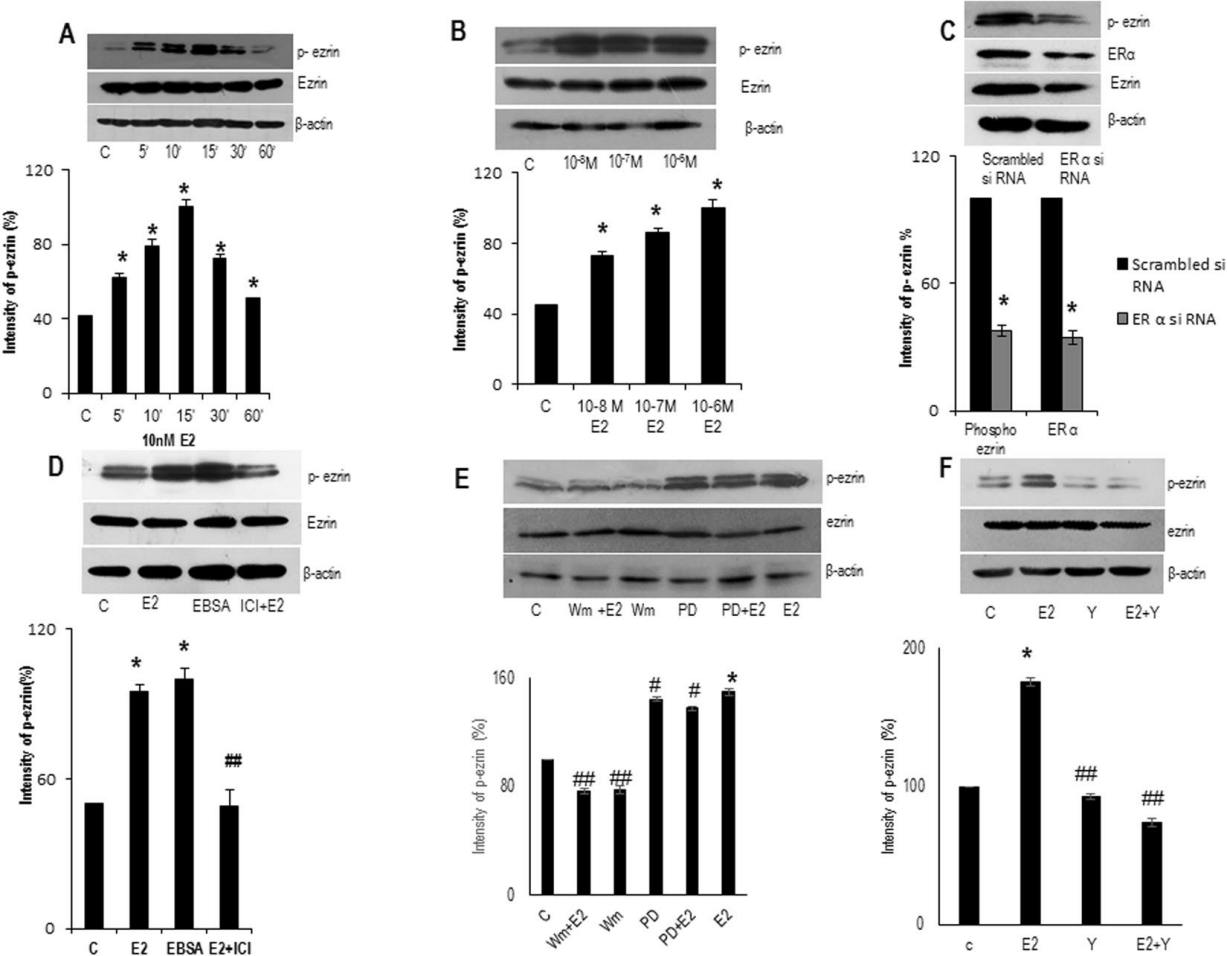
E2 rapidly phosphorylates (T567) ezrin a time and dose dependent manner. (**A**) Western blot analysis showing time dependent phosphorylation (T567) of ezrin in ERα positive FTC-133 cells after treatment with E2 (10 nM) for different time points (5′, 10′, 15′, 30′ 60′) of one hour. (**B**) Western blot showing expression of phospho-ezrin in FTC-133 cells were treated with increasing concentrations of E2 (10^−8^ M, 10^−7^ M, and 10^−6^ M) for 15 minutes. (**C**) FTC-133 cells were exposed to ERα siRNA and investigated the expression phospho-ezrin in ERα suppressed cells. (**D**) Western blot showing FTC cells treated with E2 (10 nM), EBSA (10 nM) and ICI (1 µM) in the presence of E2 (10 nM) for 15 minutes. (**E**) Western blot showing expression of phospho-ezrin in FTC-133 cells exposed to E2 (10 nM), MEK inhibitor PD 98059 (20 µM) and PI3K inhibitor wortmannin (30 nM) in presence or absence of E2. (**F**) Western blot showing expression of phospho-ezrin in FTC- 133 cells treated with E2 (10 nM) and ROCK-2 inhibitor Y- 27632 (10 µM) in presence or absence of E2. Densitometry values of phospho-ezrin is adjusted and normalized to that of ezrin intensity. All the experiments were repeated three times and the representative images are shown. Statistical significance was calculated using t-test *p < 0.05 vs. control; ^#^p < 0.05, ^##^p < 0.05 vs. E2 respectively.

### E2 enhanced the activation of ezrin via ERα

To check E2 is responsible for the rapid activating phosphorylation of ezrin, we treated FTC-133 cells with E2 (10 nM) for different time points of one hour. Treatment with E2 (10 nM) resulted in rapid phosphorylation of Thr567 residue of ezrin. This process was time dependent, transient being maximal at 10–15 minutes and showed a tendency to reverse back almost to the basal level (Fig. 3A). Meanwhile, total ezrin protein remained the same (Fig. 3A). Meantime Thr567 phosphorylation was found to be increasing with increasing concentrations of E2 in FTC cells (Fig. 3B).

To identify the ER subtype responsible for activation of ezrin, FTC-133 cells were treated with E2 (10 nM), ERα agonist PPT (10 nM) and ERβ agonist DPN (10 nM) for 15 minutes. Similar to the above result, the activation of ezrin was more in cells treated with E2 and ERα agonist (Supplementary, Fig. S1A). We investigated the expression of phosoho-ezrin in ERα silenced cells. ERα knockdown abolished the activation of ezrin (Fig. 3C). To confirm that the activation of ezrin occurs via an extra-nuclear pathway, FTC-133 cells were pre-treated with an inhibitor of translation, cycloheximide (50 µM) and then treated with E2 (10 nM) for 15 minutes, analysed the phosphorylation status of ezrin. Treatment with E2, indeed resulted in the activation of ezrin even in presence of cycloheximide (Supplementary, Fig. S1B) suggesting that the activation process does not involve *de novo* protein synthesis. Furthermore, treatment of FTC cells with estradiol-bovine serum albumin conjugate (EBSA) (10 nM), a membrane impermeable form of E2 (Fig. 3D), also resulted in rapid activation of ezrin indicating that extra-nuclear signalling of E2 is possibly a membrane initiated one.

### Role of PI3K/AKT and ROCK-2 in signalling from  ERα to ezrin

To identify the kinase candidates involved in the rapid activation of ezrin at Thr567, we screened pharmacological inhibitors of kinases to block extra-nuclear pathways. The inhibitors like PD98059 to block the mitogen activated kinase kinase (MEK), wotmannin to block the Phosphoinositde 3-kinase (PI3K) and Y27632 to block ROCK-2 were used. Pre-treatment with wortmannin (30 nM) for 30 minutes abolished the phosphorylation of ezrin (Fig. 3E) and exposure of cells to PD98059 (20 µM) did not change the phosphorylation (Fig. 3E). The modulation of PI3K in phosphorylation of ezrin was confirmed by inhibiting the E2 induced Akt phosphorylation using wortmannin to affirm the pathway (Supplementary, Fig. S1C). To further demonstrate the kinases responsible for the phosphorylation of ezrin, we used selective inhibitor of ROCK-2. Following the pre-treatment with Y-27632 (10 µM) for 30 minutes, the expression of phospho-ezrin was markedly diminished (Fig. 3F). The rapid effects of inhibitors on its phosphorylation may be explained by the rapid turnover of ezrin specific phosphatase following the action of inhibitors^22^. To confirm this, we used ROCK-2 siRNA to knock down the expression of ROCK-2. Exposure to ROCK-2 siRNA significantly reduced the expression of phosphorylated ezrin (Supplementary, Fig. S2A). This data suggests that PI3K and ROCK-2 are among the kinases responsible for the activation of ezrin FTC cells.

### Knockdown of ezrin decreased E2 induced migration and invasion of cells

**Figure 4:**
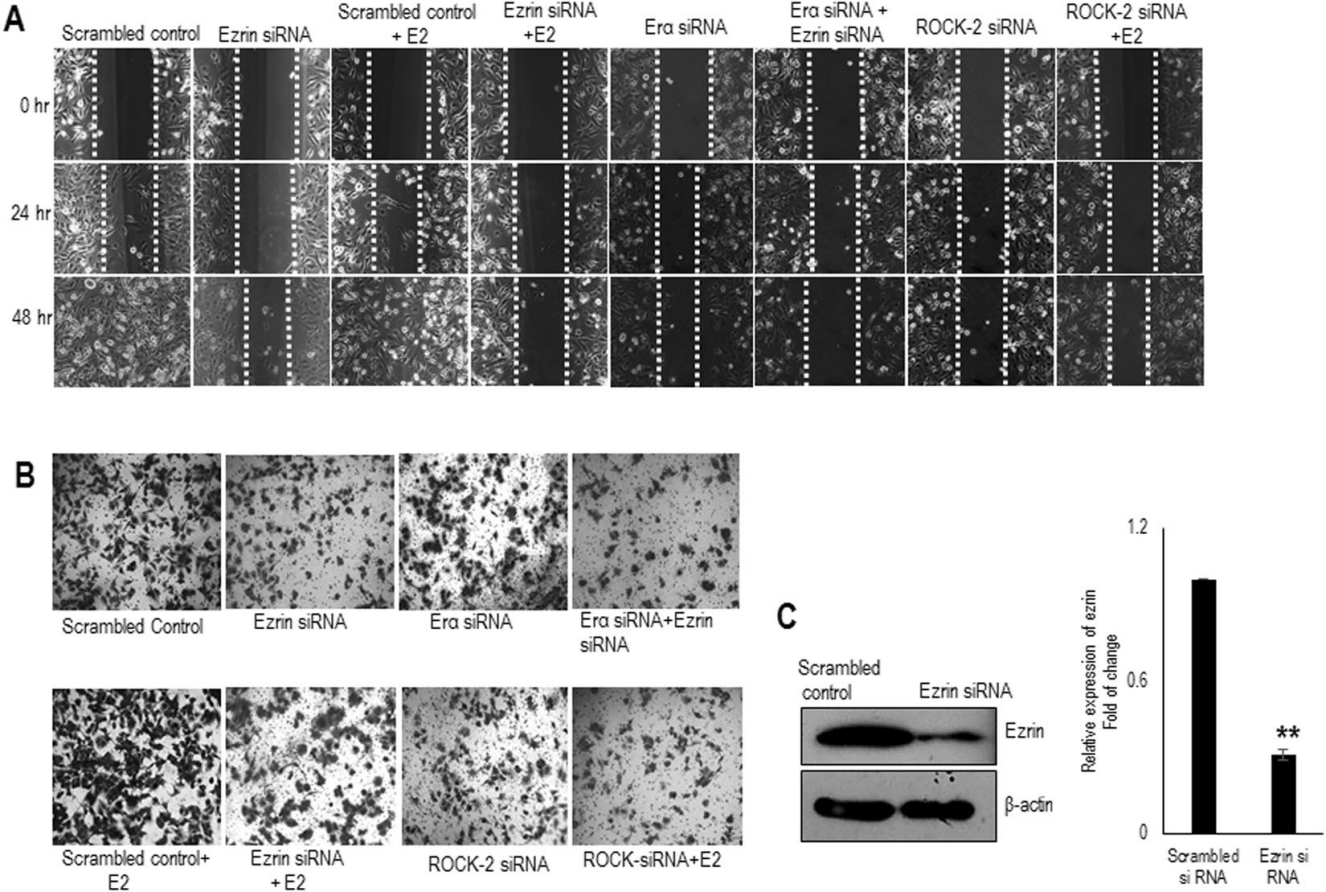
Knock down of ezrin and inhibition of phosphorylation decreases follicular cancer cell migration and invasion. (**A**) Nearly confluent FTC-133 cells were wounded by using a 0.1–10 µl tip and cells were exposed to ezrin siRNA, ROCK-2 siRNA in presence or absence of E2 (10 nM), ezrin siRNA in combination with ERα siRNA. Images of denuded areas were taken at 0, 24 and 48 hours respectively. (**B**) Invasive potential of ezrin was analysed by using matrigel invasion assay. Ezrin silenced FTC cells in presence of E2 (10 nM), ezrin and ERα silenced FTC cells, ROCK-2 silenced FTC cells were seeded in a transwell invasion chamber. Representative images of the cells invaded through the membrane are shown in the figure. (**C**) Western blot showing expression of ezrin in FTC cells exposed to ezrin siRNA. β-actin is selected as the loading control. Data is presented as mean ± standard deviation from 3 independent experiments. Statistical significance was calculated using t-test. *p ≤ 0.05, **p ≤ 0.01 vs. control.

Ezrin plays a pivotal role in migration and invasion of cells^23^. To evaluate if ezrin is implicated in E2 induced migration, we investigated the migratory ability of ezrin silenced FTC cells. For this, ezrin was functionally silenced using ezrin siRNA (Fig. 4C). FTC-133 cells were wounded, and the migratory ability was analysed at various time points with or without the treatment of E2 (10 nM). In ezrin silenced cells, the migration of cells to heal the wound was significantly delayed until 48 hours compared to that in the scrambled siRNA transfected cells supporting an antimigratory phenotype (Fig. 4A). E2 enhanced the migration of cells significantly and this enhancement in migration was reduced by silencing ezrin (Fig. 4A). To explore the biological significance of PI3K and ROCK-2 regulation on E2 induced ezrin activation, we examined the effect of inhibitor wortmannin (30 nM) and ROCK-2 siRNA on cell motility with E2 (10 nM). Blocking PI3K or ROCK-2 eventually reduced the E2 induced migration (Fig. 4A, Supplementary, Fig. S2B). At the same time, suppressing ERα in ezrin silenced cells also reduced the E2 induced migration of cells (Fig. 4A) suggesting that E2 induced ezrin expression occurs via ERα. To confirm the antimigratory role of ezrin silenced cells, invasion assays were also performed using Matrigel Invasion Chamber. The invasion of cells induced by E2 was blocked by knocking down ezrin (Fig. 4B) and by the inhibitor of PI3K or ROCK-2 siRNA (Fig. 4B, Supplementary, Fig. S2C). The invasion of ezrin silenced cells were further reduced by silencing ERα as in the previous case (Fig. 4B).

### Ezrin silencing altered the Epithelial – Mesenchymal Transition (EMT) related protein levels

**Figure 5:**
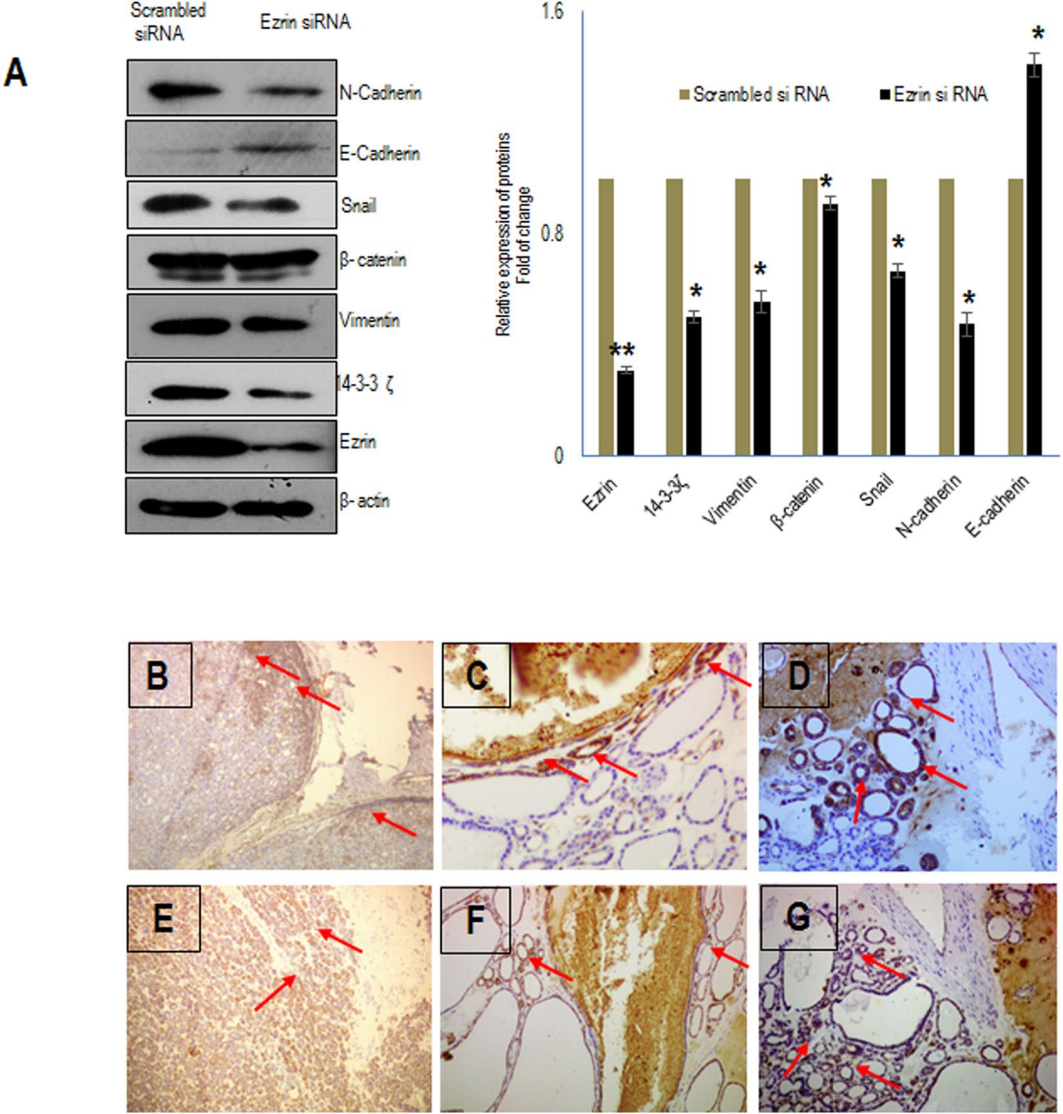
Ezrin silencing altered epithelial to mesenchymal transition proteins. (**A**) Western blot showing expression of EMT proteins E-Cadherin, N-Cadherin, vimentin, β-Catenin, snail and 14-3-3 ζ in ezrin silenced cells. Representative micrographs showing intense expression of phospho-ezrin in follicular cells adjacent to the capsule (**B**,**D**) and the blood vessel (**C**,**D**). Images showing the same pattern of immunostaining of ezrin in the follicular cells adjacent the capsule (**E**,**G**) and the blood vessel (**F**) of follicular thyroid carcinoma lesions.

Our data suggests that ezrin knockdown attenuated the cell migration and invasion. Therefore, we examined the molecular changes in ezrin silenced cells. We observed that silencing the function of ezrin in FTC cells altered EMT proteins like E-cadherin, N-cadherin, vimentin, snail and 14-3-3 ζ. We observed an upregulation of E-cadherin and downregulation of proteins like N-cadherin, vimentin, β-catenin, snail (Fig. 5A). Since 14-3-3 ζ is found to be overexpressed in several cancers and it has been shown that 14-3-3 ζ mediated membrane ruffling is ezrin dependent^24^, we analyzed the expression of 14-3-3 ζ protein in the ezrin down-regulated cells. Ezrin silencing abolished the expression of 14-3-3 ζ (Fig. 5A). Immunohistochemical analysis of some FTC lesions revealed that the expression of phospho-ezrin (T567) was only observed in follicular cells adjacent to infiltrating capsule (Fig. 5B,D) and blood vessels (Fig. 5C,D). Rest of the follicular area was unstained since there might be a loss of phospho-ezrin in later stages of metastasis, reexpressing only in central portions of expanding metastatic lesions and invasive front suggesting the pivotal role of phospho-ezrin in hematogenous and lymphatic metastasis which aids in invasion. Similarly, the expression of ezrin also displayed the same pattern in those follicular lesions (Fig. 5E–G). These results were in accordance with the cellular migratory ability measured by wound healing and transwell invasion assay.

### Ezrin and phospho-ezrin was over-expressed in FTC and FVPTC

The number of lesions taken for the study are represented in Table 1. The age, sex, reproductive age (18–45 years females), advanced reproductive age (>45 years Females) and histological frequency of ezrin and phospho-ezrin are represented in the Table 2. The epidemiological data revealed that majority of patients from which thyroid lesions obtained were females of reproductive age. In the case of FTC, out of 10 patients, 6 were females and 4 belonged to reproductive age. In the case of FA, out of 6 patients, 4 belonged to females and 2 belonged to reproductive age (Table 2). Among FVPTC, all the lesions were from females and among them, 6 belonged to reproductive age. Among PTC, out of 10 patients, 8 belonged to females and among them, 6 belonged to reproductive age. In the case of goiter all the 3 lesions were from females and among them, 2 belonged to reproductive age. This data affirms the fact that thyroid disorders are more prevalent in menstruating females.

**Table 1:** Number of different thyroid lesions.

Thyroid tissue lesions	Number
Follicular adenoma	6
Follicular thyroid carcinoma (FTC)	10
Papillary thyroid carcinoma (PTC)	10
Follicular variant of papillary thyroid carcinoma (FVPTC)	9
Multinodular goiter	3
Total	38

**Table 2:** Age distribution, sex and histological frequency of patients.

Histopathlogy Report	Sex frequency		Reproductive age (18–45 years females)	Advanced reproductive age (>45 years)	Histology frequency ezrin	Histology frequency of phospho-ezrin
	F	M				
FTC	6	4	4	2	10	10
FA	4	2	2	4	6	0
FVPTC	9	0	6	3	9	9
PTC	8	2	6	4	10	0
Goitre	3	0	2	1	0	0

**Figure 6:**
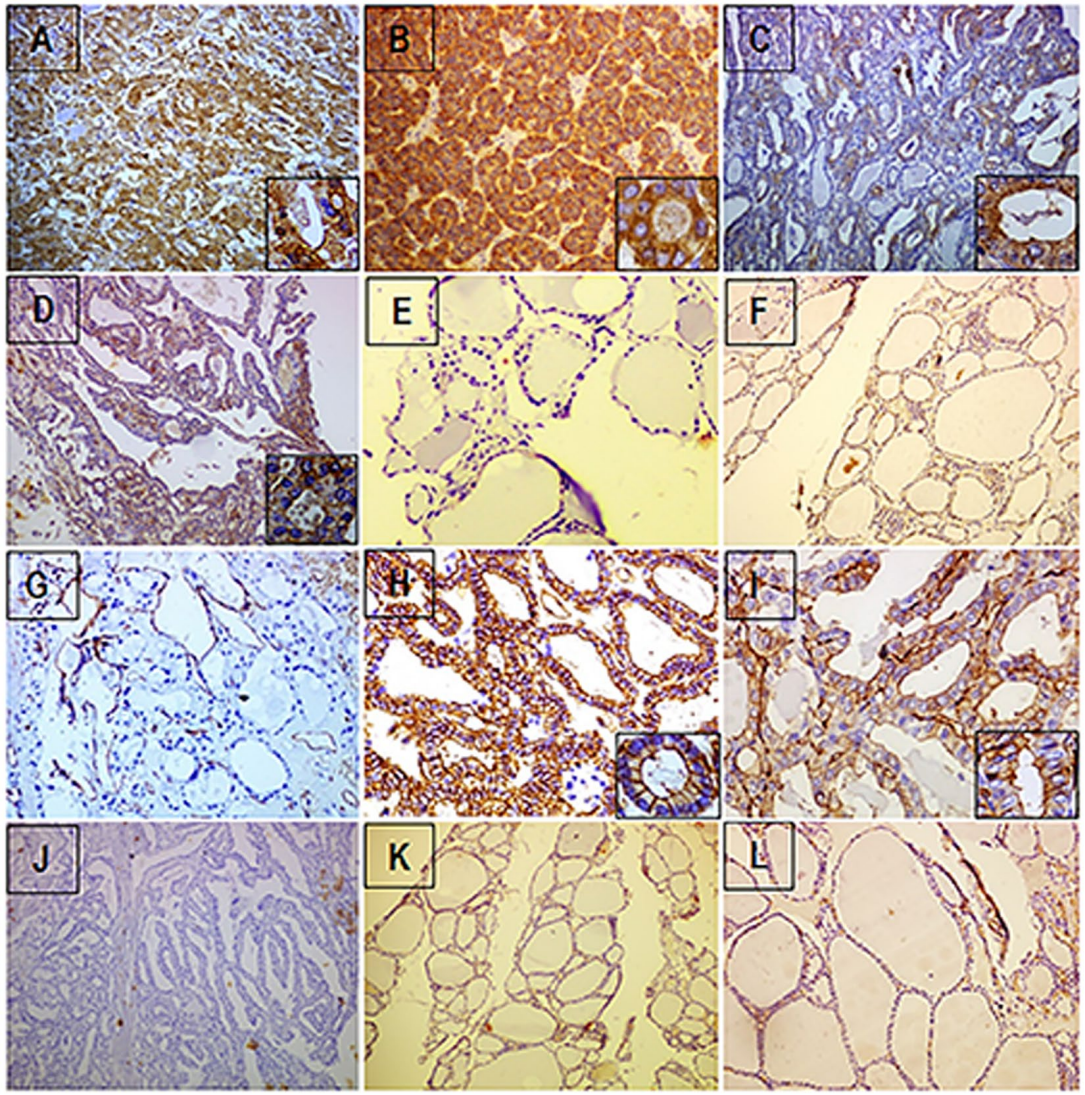
Ezrin is overexpressed in tumour cells. Representative micrographs of immunohistochemical staining patterns of ezrin in different thyroid lesions. (**A**) Follicular adenoma. (**B**) Follicular thyroid carcinoma (**C**) Follicular variant of papillary thyroid carcinoma. (**D**) Papillary thyroid carcinoma. (**E**) Adjacent benign follicular tissues and (**F**) goitre lesions. Representative micrographs showing immunohistochemical staining pattern of phospho-ezrin in (**G**) follicular adenoma (**H**) follicular thyroid carcinoma lesions (**I**) Follicular variant of papillary thyroid carcinoma. (**J**) Papillary thyroid carcinoma lesion, (**K**) adjacent benign tissue and (**L**) goitre lesion.

**Figure 7:**
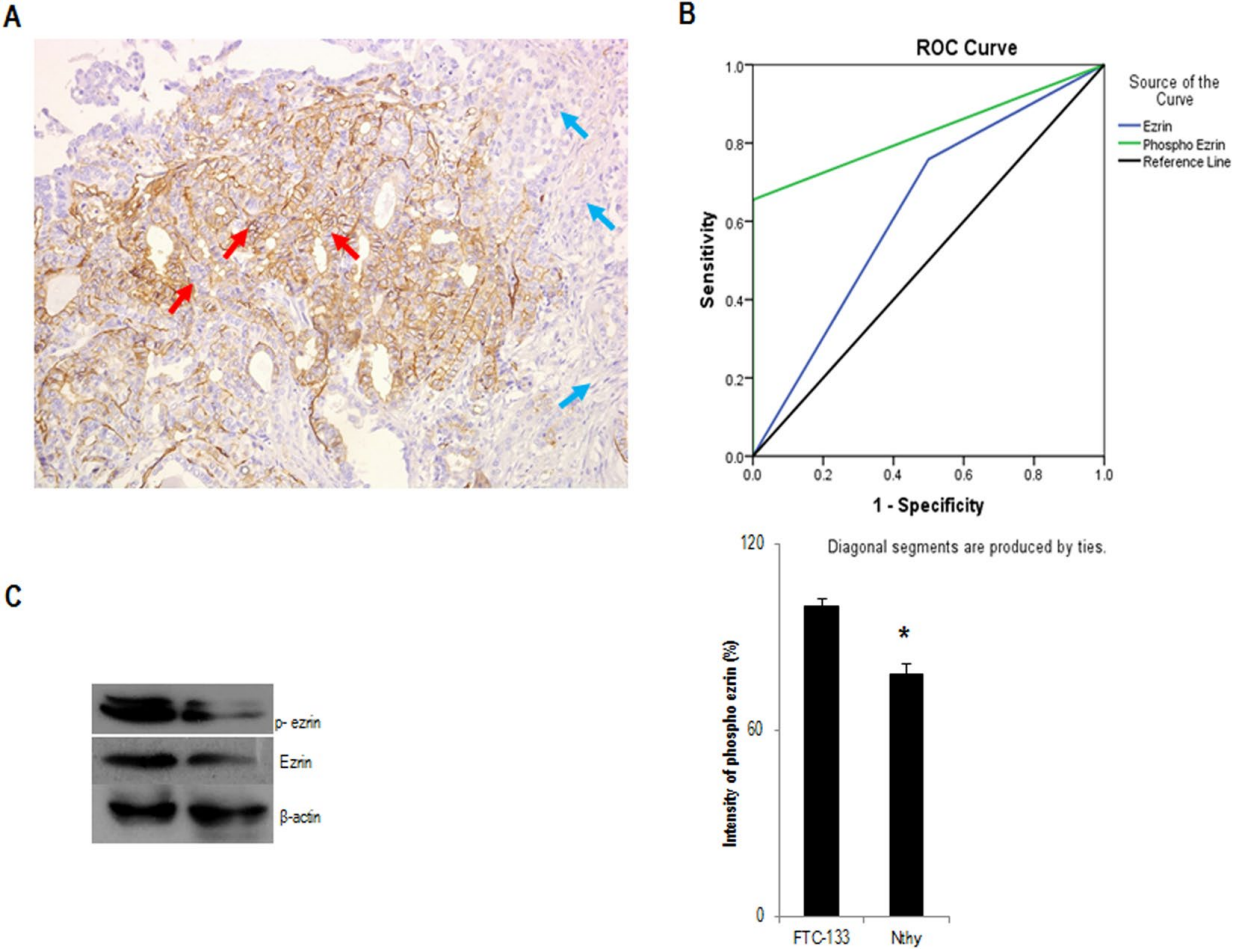
Phospho-ezrin is over expressed in FTC-133 cells. (**A**) Representative micrograph showing expression of phospho-ezrin in follicular variant of papillary thyroid carcinoma; red arrows indicate positive staining of phospho-ezrin in follicular area of FVPTC and blue arrows indicate negative staining in papillary area of FVPTC. (**B**) Representative image of ROC curve for the histological association of all thyroid carcinomas and follicular adenoma. (**C**) Western blot showing expression of phospho-ezrin in FTC-133 and Nthy cells. β-actin is selected as the loading control. Data is presented as mean ± standard deviation from 3 independent experiments. Statistical significance was calculated using t-test. *p ≤ 0.05, **p ≤ 0.01 vs. control.

Immunohistochemical analysis revealed that ezrin immunoexpression was found predominantly in cytoplasm of all thyroid lesions except goitre. In FTC, intense expression of ezrin was observed in both cytoplasm and membrane (Fig. 6B) when compared to the moderate cytoplasmic staining of ezrin in follicular adenoma (Fig. 6A). Whereas adjacent benign tissue was negatively stained for ezrin (Fig. 6E) indicating it significant role in invasion in FTC. In FVPTC, moderate to intense staining of ezrin was observed in cytoplasm, as well as membrane (Fig. 6C). In the case of PTC, only mild to moderate expression of ezrin was observed (Fig. 6D). Staining was predominantly cytoplasmic in most of the cells in PTC. Goiter lesions were negatively stained for ezrin (Fig. 6F). In case of phospho-ezrin, it was negatively stained in FA (Fig. 6G). Whereas in FTC, all the lesions were intensely stained and membranous staining was observed in the majority of follicular cells (Fig. 6H). Adjacent benign cells were negatively stained for phospho-ezrin as they are not invasive (Fig. 6K). In FVPTC, phospho-ezrin was intensely stained only in follicular regions of all the lesions (Figs 6I, 7A). Membranous staining was observed in follicular cells (Fig. 6I). Rest of the papillary area was unstained for phospho-ezrin (Fig. 7A). In PTC all the lesions were negatively stained for phospho-ezrin (Fig. 6J). The ‘H’ score for ezrin and phospho-ezrin of different thyroid lesions is represented in the Table 3. The ‘H’ score for both markers was observed to be higher in FTC and FVPTC indicating that both markers are intensely expressed in FTC and FVPTC. All the goitre lesions were negatively stained for phospho-ezrin (Fig. 6L).

**Table 3:** Mean ‘H’ score for ezrin and phospho-ezrin of different thyroid lesions.

Thyroid lesions	Mean ‘H’ score for ezrin	Mean ‘H’ score for p-ezrin
Follicular adenoma (FA)	110	0
Follicular thyroid carcinoma (FTC)	160	160
Papillary thyroid carcinoma (PTC)	110	0
Follicular variant of papillary thyroid carcinoma (FVPTC)	150	150

### Histological association between all thyroid carcinomas and benign FA with respect to phospho-ezrin is highly significant

The histological correlation of each type of carcinoma and FA was assessed by Fisher’s exact test. We compared the ‘H’ scores of all carcinomas with FA. ‘H’ score ≥ 100 was considered as the cut off value for each marker. The histological association between FA and all carcinomas with respect to ezrin is shown in Table 4. From the table, it is evident that in case of FTC, all the tissues taken for the study were positive for ezrin. In the case of FVPTC, out of 9 lesions, only 6 lesions were positive and in PTC, out of 10 lesions, only 6 were positive for ezrin. But this histological association was not statistically significant (p = 0.079) since half of the FA lesions taken were positive for ezrin. The histological association between all carcinomas and FA with respect to phospho-ezrin is represented in Table 5. From Fisher’s exact test, it is revealed that all the FTC and FVPTC lesions taken for the study were positive for phospho-ezrin. All FA and PTC lesions were negative for phospho-ezrin. Since, there was a higher expression of phospho-ezrin in FTC and FVPTC and absence of expression of the marker in FA, the above histological association was highly significant (p = 0.0001) (Table 5).

**Table 4:** Histological association between follicular adenoma and all thyroid carcinomas with respect to ezrin (Fisher’s exact test).

Marker	Histology				Total	P value
	Follicular thyroid carcinoma	Follicular adenoma	Follicular variant of papillary thyroid carcinoma	Papillary thyroid carcinoma		
Ezrin ≤100 count % within ezrin	0 0.0%	3 30.0%	3 30.0%	4 40.0%	10 100%	0.079
Ezrin >100 count % within ezrin	10 40.0%	3 12.0%	6 24.0%	6 24.0%	25 100%	
Total % within ezrin	10 28.6%	6 17.1%	9 25.7%	10 28.6%	35 100%

**Table 5:** Histological association between follicular adenoma and all thyroid carcinomas with respect to phospho-ezrin (Fisher’s exact test).

Marker	Histology				Total	P value
	Follicular thyroid carcinoma	Follicular adenoma	Folliular variant of papillary thyroid carcinoma	Papillary thyroid carcinoma		
Phospho-ezrin ≤100% within phospho-ezrin	0 0.0%	6 37.5%	0 0.0%	10 62.5%	16 100%	0.0001
Phospho-ezrin >100% within phospho-ezrin	10 52.6%	0 0.0%	9 47.4%	0 0.0%	19 100%	
Total count % within phosho-ezrin	10 28.6%	6 17.1%	9 25.7%	10 28.6%	35 100%	

Based on the ‘H’ score of different thyroid lesions, we calculated the sensitivity and specificity for the histological association between each thyroid carcinoma and FA. Thyroid lesions with ‘H’ score ≥100 were taken as positive and invasive. In the first case, we correlated the histology between FTC and FA for ezrin and phospho-ezrin. We observed that the sensitivity for both the markers ezrin and phospho-ezrin was 100% (Table 6). A test with 100% sensitivity correctly identifies all lesions (true positives) positive for both markers and are invasive. Specificity, the true negative rate (‘H’ score ≤100) was only 50% for ezrin (Table 6). 50% specificity correctly identifies 50% of lesions negative for the marker and not invasive, but 50% lesions were incorrectly identified as test positive (false positives). In this test, false positives were FA lesions with an ‘H’ score ≥100 and not invasive. In the case of phospho-ezrin, the specificity was observed to be 100% (Table 6). Therefore 100% specificity correctly identifies all lesions that are not invasive. None of the FTC lesions were negative for phospho-ezrin (‘H’ score ≤00). So the negativity of the marker phospho-ezrin determines the lesions that are not invasive. Moreover, the false negative rate was zero. That is, none of the lesions had ‘H’ score ≤100 and were invasive. The positive predictive value (PPV) is the proportion of positive test that is the true positives and represents the presence of a condition. Negative predictive value (NPV) is the proportion of negative test that is true negatives and represents the absence of disease. In this test, the PPV for the marker ezrin is 90.91% (Table 6). Whereas the PPV for phospho-ezrin is 100% indicating all the test positives are true positives and the test is ideal and good as gold standard. The negative predictive value (NPV) for ezrin and phospho-ezrin is 100% (Table 6) indicating all the negative tests are true negatives for both the markers and the test is ideal and good as gold standard. Diagnostic accuracy of a test is the ability to differentiate lesions that are invasive and not invasive correctly. In this test, 100% sensitivity and specificity led to the diagnostic accuracy of 100% (Table 6) for the marker phospho-ezrin indicating that that the test is excellent for this marker (p = 0.0001). In case of histological association between FVPTC and FA, again we observed 100% sensitivity and specificity led to 100% diagnostic accuracy for phospho-ezrin (p = 0.001) (Table 7). In case of histological association between PTC and FA, the sensitivity and specificity of ezrin was significantly less and for phospho-ezrin, it was 0 and 100%. Both markers were insignificant for this association (Table 8). The overall sensitivity, specificity of ezrin and phospho-ezrin for the association between all thyroid carcinomas and FA was calculated. The sensitivity and specificity for the marker ezrin was observed to be 67.99% and 50%. The sensitivity and specificity for phospho-ezrin were 79.5% and 100% which was considerably higher than that for ezrin (Table 9). The higher sensitivity and specificity of phospho-ezrin led to the diagnostic accuracy of 81.48% which enables us to suggest phospho-ezrin as a diagnostic marker.

**Table 6:** Histological association between Follicular thyroid carcinoma and follicular adenoma.

Biomarker	Sensitivity (%)	Specificity (%)	PPV (%)	NPV (%)	Diagnostic accuracy (%)	P value
Ezrin	100	50	76.9	100	81.25	0.036
Phospho-ezrin	100	100	100	100	100	0.0001

**Table 7:** Histological association between Follicular variant of papillary thyroid carcinoma and follicular adenoma.

Biomarker	Sensitivity (%)	Specificity (%)	PPV (%)	NPV (%)	Diagnostic accuracy (%)	P value
Ezrin	66.7	50	66.7	50	60	0.622
Phospho-ezrin	100	100	100	100	100	0.001

**Table 8:** Histological association between papillary thyroid carcinoma and follicular adenoma.

Biomarker	Sensitivity (%)	Specificity (%)	Positive Predictive value PPV (%)	Negative Predictive Value NPV (%)	Diagnostic accuracy (%)	P value
Ezrin	60	50	66.7	42.9	56.3	1
Phospho-ezrin	0	100	0	37.5	37.5	0

**Table 9:** Histological association between all thyroid carcinomas and follicular adenoma (FA).

Biomarker	Sensitivity (%)	Specificity (%)	Positive Predictive value (PPV)	Negative Predictive Value (NPV)	Diagnostic accuracy (%)
Ezrin	67.9	50	80.2	30.0	60.03
p ezrin	79.5	100	37.5	100	81.48

### Receiver operating characteristic (ROC) curve for histological association between all thyroid carcinoma (PTC) and follicular adenoma (FA)

The ROC curve for the association between all thyroid carcinoma and FA is represented in the figure (Fig. 7B). The blue colored curve on the graph represents the curve for ezrin. The sensitivity for ezrin in this association is 67.9%  and specificity is 50%. Therefore, the 1-specificity, sensitivity pair for ezrin is (0.5,0.679). The green curve represents the curve for phospho-ezrin. The sensitivity and specificity for phospho-ezrin is 79.5% and 100%. Therefore, the 1-specificty, sensitivity pair for phospho-ezrin is (0,0.795 ), From the graph, the area under the curve (AUC) for phospho-ezrin is 0.828 and for ezrin is 0.629 respectively (Fig. 7B). It is evident that AUC for phospho-ezrin is higher and significant compared to the AUC for ezrin. Therefore, phospho-ezrin can be suggested as a marker to discriminate between lesions that are invasive and not invasive. The ROC curve for each histological association is represented in the figure (Supplementary, Fig. S3A–C). The area under the curve for phospho-ezrin for the association between FTC and FA, FVPTC and FA was 1, which renders 100% accuracy for the marker in a diagnostic test. Our *in vitro* data also supported the fact that phospho-ezrin is over expressed in FTC cells when compared to the Nthy cells (Fig. 7C).

## Discussion

In the present study, presence of ERα was only observed in PTC (Fig. 1B) and FVPTC (Fig. 1C) lesions. We could not observe the presence of ERα in FTC lesions. Those tissues might not be estrogen responsive or there might be a chance of loss of receptors which are very sensitive due to the technical reasons like tissue processing and long term storage. Estrogen metabolism stimulates the cell mobility in many ER positive cells, later to invade and metastasise into surrounding blood and lymphatic vessels thus contributing to a devastating disease^25^. In agreement to the above fact, we observed that E2 has a promigratory effect by inducing the expression of ezrin in ERα positive thyroid cells (Fig. 2A) whereas ERα silenced FTC-133 cells (Fig. 2F) displayed a reduced expression of ezrin, confirming a critical regulatory role of E2 on ezrin via ERα. These results in thyroid cancer cells were compatible with the previous reports showing that estrogen enhanced the growth and metastatic phenotype of ovarian, breast and endometrial carcinoma cells^20,21^. The *ex vivo* analysis also revealed that majority of patients from which thyroid lesions obtained were females of reproductive age (Table 2).

E2 can cause rapid changes in most of the signalling molecules especially like phosphorylation, rapid increase in secondary messengers and calcium uptake^26^. Ezrin is present in cytoplasm in dormant conformation. Phosphorylation of specific residues in ezrin followed by the translocation to cytoplasmic membrane where the N-terminal end associates with either the plasma membrane or the integral membrane proteins, whereas the C- terminal associates with F- actin, and functions as a dynamic membrane cytoskeletal linker^27^. Sex steroids, particularly estrogen can rapidly cause the membrane translocation of ezrin and ERα and formation of membrane ruffles as well as pseudopodia in endothelial and breast cancer cells^28,29^. We observed that E2 rapidly phosphorylates Thr567 and this action was transient, a characteristic of non - genomic action (Fig. 3A). Although the membrane ERs are identified in many different tissues^30^, the specific location of ER for the non-genomic process is not well known. This study revealed that there might be a chance of membrane initiated signalling by using a membrane impermeable conjugate of E2, such as EBSA assuming it is an E2 mimetic. EBSA mimicked the effect of E2 by rapidly phosphorylating ezrin (Fig. 3D). In non-genomic action, translation and transcription processes are unlikely, which is characterised by rapid time lapse and thus insensitive to transcription and translation inhibitors^31^. In accordance to that we found that rapid ezrin activation by estrogen was not blocked by cycloheximide, a protein translation inhibitor (Supplementary, Fig. S1B).

The extra-nuclear activation of ezrin is achieved by recruitment of kinase cascades and interaction of these kinases with membrane ER^32^. Based on the inhibitor study, the chemical-based inhibition of PI3K resulted in significant reduction in the E2 induced phosphorylation of ezrin (Fig. 3E). It is documented that E2 induced PI3K activation resulted in the activation of ROCK-2 cascade eventually phosphorylating ezrin. ROCK proteins are involved in different cellular activities like actin cytoskeleton organization, cell adhesion and motility^33^. The present study revealed that the targeted inhibition of ROCK-2 attenuated the phosphorylation of ezrin when FTC cells exposed to ROCK-2 inhibitor and siRNA (Fig. 3F, Supplementary, Fig. S2A). Consequently, it is possible that E2 indirectly phosphorylates ezrin Thr567 via PI3K/AKT/ROCK-2 cascade. Other signalling cascades phosphorylating ezrin in thyroid carcinoma during estrogen exposure has to be identified.

It has been reported that dominant negative mutants of RNA interference studies reversed the metastatic behaviour of breast cancer cell lines^34^. In accordance to it, ezrin silenced FTC cells displayed an antimigratory phenotype (Fig. 4A) and it greatly reduced the invasiveness of the cells (Fig. 4B). This effect was not rescued by E2 treatment (Fig. 4A) confirming the stimulatory effect of E2 on ezrin via ERα. In our study, we acquainted the effects of non-genomic actions in migration and invasion by hindering the E2 induced activation of ezrin by inhibitors (Fig. 4A, Supplementary, Fig. S2B,C). The effects of rapid processes induced by E2 in physiological processes are sparsely reported, and the confounding influence of these rapid processes is elusive. Moreover, the estrogen dendrimer conjugate (EDC) a selective ER modulator (SERM) which preferentially activated extra nuclear pathways and induced reendothelization and neointimal development in mice after an injury^24^.

Since our results revealed that ezrin silencing induced antimigratory effects in FTC cells, we examined the expression of certain critical molecules in cell adhesion process. In the present study, the expression of E-cadherin was elevated and the expression of snail, N-Cadherin and vimentin were diminished in ezrin down regulated cells (Fig. 5A) suggesting that the localization of these proteins are regulated by ezrin. We observed a cadherin switch, that is the decrease in the expression of N–cadherin and increased expression of E–cadherin in ezrin down regulated cells (Fig. 5A) suggesting that ezrin overexpression makes the cells more vulnerable to EMT.

The clinical applications or the potential of ezrin and phospho-ezrin were studied in thyroid lesions. As an invasive protein, the impact of ezrin in invasion of thyroid carcinoma is minimally investigated. Immunohistochemical analysis revealed that the staining of ezrin was predominantly cytoplasmic in FA (Fig. 6A), whereas both cytoplasmic and membranous staining of ezrin was observed in all thyroid carcinoma lesions except PTC indicating its translocation from cytoplasm to the membrane during cancer progression. Many studies suggest that cellular redistribution of ezrin is strongly correlated with tumorigenesis^35^. Surprisingly, in case of phospho-ezrin, we observed intense membranous staining in FTC and FVPTC and it was completely absent in FA and PTC. The distant metastasis of PTC is uncommon compared to other histotypes of thyroid cancer. It is typified by low rates of mortality, aggressiveness and 100% survival rate was observed for stage I disease^36^. To find out the exact reason, we may have to extend the studies in a greater number of PTC lesions.

We were so overwhelmed to see the results that it has thrown light on the dilemma of differentiation of follicular neoplasms. Thyroid nodules showing follicular morphology include FA, FTC and FVPTC. Cytologic features overlap among these tumors and the definite diagnosis to differentiate these entities is problematic^37^. Differential diagnosis between FTC and FA is based on the presence of capsular and vascular invasion, distant or nodal metastasis in FTC^38,39^. Diagnosis based on cytological features alone has always been challenging to cytopathologists and clinicians. Most of the times, FNAC is also not consistent and leads to false positive results which becomes non-diagnostic. So, it requires a diagnostic lobectomy to confirm the neoplasm followed by total thyroidectomy and even after surgery the differential diagnosis is problematic^40^. From our study, it has been found that phospho-ezrin was intensely expressed in FTC and was completely absent in FA (Fig. 6G,H). The histological association between the two revealed that the marker phospho-ezrin has high sensitivity (100%) and specificity (100%) which lead to 100% diagnostic accuracy for the test (Table 6). Moreover, from the ROC curve, the accuracy for phospho-ezrin was observed to be 1, in which the test is excellent for the marker phospho-ezrin (Supplementary, Fig. 3A). Therefore, in the dilemma of differentiation of the two, it was sufficient enough for us to suggest phospho-ezrin as a diagnostic marker to differentiate FTC from FA. Similarly, FVPTC cytologically almost completely comprises of follicular morphology with nuclear features of PTC^41^. The encapsulated FVPTC behave indolently, similar to FA in which the accurate diagnosis is again challenging for clinicians^41^. We observed that phospho-ezrin was intensely stained in all FVPTC lesions. From the histological association between FVPTC and FA, the sensitivity and specificity of this association was 100% which led to the diagnostic accuracy of 100% to differentiate the two (Table 7). Moreover, from ROC curve, the accuracy for phospho-ezrin was observed to be 1 which means the test is excellent for the marker phospho-ezrin. Our data revealed that phospho-ezrin was intensely expressed in the follicular area of FVPTC and completely absent in FA (Fig. 6G,I). Therefore phospho-ezrin can be suggested a diagnostic marker to differentiate FVPTC from FA.

## Methods

### Clinical samples and immunostaining

This study was conducted in archival thyroid tissues obtained from patients attending thyroid clinic of Regional Cancer centre, Thiruvananthapuram. ‘Informed consent’ was obtained from the patients for study participation. A total of 38 samples comprising six FA, ten FTC, ten PTC, nine FVPTC and three multinodular goitre were procured from patients underwent surgery. Due to the lesser availability of FA lesions, we were forced to perform our experiments within the six available tissue samples. Formalin fixed paraffin embedded tissue samples were serially cut at 4 micron thickness using microtome (Leica TP1020). Immunohistochemistry was performed by standard avidin-biotin complex method. Biocare medical Mach 4 Universal HRP-Polymer Detection System was used in current study. Mouse monoclonal antibody against ezrin, (1:100), ER α (1:100, Santa Cruz Biotechnology) and rabbit polyclonal antibody to phospho-ezrin-T567 (1:100, Abcam) were used. Sections were incubated with secondary antibody for 30 minutes. The DAB substrate detection system (Sigma-Aldrich) was used. Sections were counterstained with hematoxyline, dehydrated and mounted with coverslips. The results were scored on a semiquantitative way by using ‘H’ score method.

### Cell culture and reagents

The Human follicular thyroid carcinoma (FTC-133) and normal thyroid follicular epithelial cell line Nthy-ori3-1 (Non-tumorigenic) were obtained from Sigma Aldrich. Nthy and FTC-133 cells were cultured in Dulbecco’s Modified Eagle medium (DMEM) supplemented with 10% fetal bovine serum (FBS). At 60–70% confluence, medium was changed to hormone, growth factor and phenol red free DMEM supplemented with 5% charcoal stripped FBS (CTS) before experiments were started. Inhibitors used were added 30 minutes prior to starting the experiment. 17β–estradiol (E2), ICI-182780 (ICI), Y-27632, wortmannin and PD-98059 were from Sigma-Aldrich. 4′,4″,4″′-(4-propyl-[1H]-pyrazole-1,3,5-triyl) trisphenol 10 nM (PPT), 2,3-bis-(4–hydroxyphenyl)-propionitrile 10 nM (DPN) were purchased from Tocris Bioscience.

### Immunoblotting

Cell lysates were resolved in SDS-PAGE and transblotted onto polyvinylidene difluroide (PVDF) membrane. Primary antibodies used were phospho-ezrin (T567), E-cadherin, Snail, β-catenin, vimentin, 14-3-3 ζ, Phopho-Akt (Ser-^473^) (1:1000Cell Signalling Technology), Ezrin, ERα (1:500, Santa Cruz), β-actin 1:2000 (Sigma Aldrich). The blots were incubated overnight with primary antibodies at 4 °C. HRP conjugated anti-mouse and anti-rabbit (1:10000, Sigma Aldrich) were used as secondary antibodies. Immunoreactive proteins were detected by enhanced chemiluminescence.

### Immunocytochemistry

FTC-133 cells were seeded on coverslips in a 12 well plate at an appropriate density of 60% confluency. 24 hours later the cells were fixed with 4% paraformaldehyde. Then cells were permeabilized with 0.3% tween 20 and blocked with 1% Bovine Serum Albumin (BSA). Primary antibody for ER α (1:100, Santa Cruz) was applied to cells overnight at 4°. Then the cells were incubated with Alexa Fluor 594 conjugated anti-mouse secondary antibody (Invitrogen) 1:400 for one hour followed by Hoechst 3334 incubation to stain the DNA. Coverslips were mounted on glycerol. Confocal microscope was used to analyse the images. Images of cells expressing ER α were acquired at 20X magnification.

### Transfection experiments

FTC-133 cells were plated in 6 well plates in DMEM high-glucose containing 10% FBS before transfection. After 24 hours at 60–70% confluence, cells were serum starved in OPTI-MEM for one hour and then incubated with 6pmoles of ezrin siRNA, 7 pmoles of ER α siRNA and 6pmoles of ROCK-2 siRNA (Santa Cruz Biotechnlogy) using lipofectamine 2000in OPTI-MEM for 6 hours. It was then replaced with DMEM containing 20% FBS and further incubated for 42 hours. Whole cell extract was prepared 48 hours post transfection. Ezrin, ERα and ROCK-2 expression level were analysed by western blot.

### Cell migration assay

Migration was evaluated using wound healing assay. FTC-133 cells were grown in 6-well plates to 90% confluence in DMEM containing 10% FBS. A denuded region was created on the monolayer culture by 0.1–10 µl pipette tips. Cells were incubated in medium containing mitomycin-C, 3 µg/µl for one hour to omit the bewildering influence of cell proliferation. Cells were incubated with ezrin si RNA, ERα si RNA and ROCK-2 si RNA in OPTI-MEM and later cells were returned to DMEM containing 10%FBS to allow cells moving into the gap. Cells were incubated with appropriate concentration of 17-β estradiol and wortmannin. Cell migration was monitored for 0, 12, 24 and 48 hours and crawled cell images were captured at each time point using Olympus IX73 microscope at 10x magnification.

### Invasion assay

The chemoinvasion assay used BD BioCoat Matrigel invasion chambers with 8 µm pore inserts. Ezrin, ERα and ROCK2 silenced cells were (7 × 10^3^) seeded in transwell chambers pre-coated with ECM matrigel and DMEM containing 10% FBS is added to the lower chamber as chemoattractant. Cells were incubated with desired concentrations of E2, and wortmannin. After 24 hours of incubation, the invaded cells in the lower surface of the membrane were stained with 2% crystal violet. The stained cells were counted and photographed by an upright microscope (Nicon Eclipse Ni-E) at 10x magnification.

### Statistical analysis

Student’s 2-tailed t-test was used to compare mean values from the quantified data of western blot. Each experiment was conducted independently at least three times. In all the tests, values were statistically significant when p ≤ 0.05. *p ≤ 0.05; **p  ≤ 0.01 vs. control and ^#^p ≤ 0.05; ^##^p ≤ 0.01 vs. E2 respectively. For immunohistochemical analysis, Statistical analysis was performed by using Statistical Package for the Social Sciences, Version 11 (SPSS) software. The diagnostic accuracy of each marker was calculated by using histopathology as the gold standard. The percentage of positivity of markers in different thyroid lesions was assessed in histologic sections. Immunohistochmistry results were evaluated by a semiquantitative approach which assigns a Histoscore (‘H’ score) to tumor samples. First the slides were read on an optical microscope Nikon Eclipse Ni-E microscope and the percentage of staining intensity was recorded for each cell in a fixed field. More intense staining was given the reading 3+, moderate staining was given the value of 2+, mild staining was given the reading of 1+ and negative staining was given the reading 0. An ‘H’ score is assigned using the formula:

$$1\,\times ( \% \,{\rm{cells}}\,1\,+\,\,)+2\,( \% \,{\rm{cells}}\,2\,+\,\,)+3\,\times ( \% \,{\rm{cells}}\,3\,+\,\,)$$

For analysis, Histoscore score value ≥ 100 was considered as the cut off value for each marker. We selected FA as the gold standard and correlated the histology of all carcinomas with respect to ezrin and phospho-ezrin by Fisher’s exact test. The Gold standard is the diagnostic test that is best available under reasonable conditions. It is the most accurate test possible without restrictions. Therefore, in this test, we have chosen follicular adenoma as the ‘gold standard’. Because it is known that benign FA is not an invasive phenotype. This test is a statistical significance test used in the analysis of frequency distribution of the variables and valid for all sample size. The above validation is confirmed by calculating area under the receiver operating characteristics (ROC) curve. Based on the ‘H’ score, corresponding sensitivity and specificity of each marker were calculated. In this study, a system or test was developed such that we correlated the histology of ezrin and phospho-ezrin for all thyroid carcinomas with the histology of those markers for follicular adenoma, in order to check the invasiveness of all the thyroid carcinoma lesions taken for the study. In this case, cut-off value (H score ≥ 100) is already known and based on the cut off score the single sensitivity, 1-specificity pair was plotted on the graph. Scores were evaluated by plotting the true positive rate (Sensitivity) in function of the false positive rate (1-specificity) for the score ≥ 100. Accuracy is measured by the area under the ROC curve. Differences were statistically significant at p ≤ 0.05.

### Ethical approval and informed consent

**Approval:** Study was approved by Regional Cancer Centre Review Board (IRB)/08-2009 as well as Rajiv Gandhi Centre for Biotechnology Human ethical committee IHECNo: 6/2009, IEC/01/2015. **Accordance:** Methods were carried out in accordance with the relevant guidelines and regulations. **Informed consent:** Informed consent has been obtained from each patient and the parents or legal guardians of patients under the age of eighteen participated in this study.

## Supplementary information

Supplementary Information

## Data Availability

All data in our study are available upon request.
