# Supplementary material for: Role of phospho–ezrin in differentiating thyroid carcinoma
Source: Sci Rep. 2019 Apr 17;9:6190. doi: 10.1038/s41598-019-42612-0 (PMC6470213; doi:10.1038/s41598-019-42612-0)
Supplement: Supplementary file 1 — Supplementary Information [file 41598_2019_42612_MOESM1_ESM.docx]

**Role of phospho–ezrin in differentiating thyroid carcinoma**

Lakshmi Mohan Lathika ^1^, Jagathnath Krishna Kumarapillai Mohanan Nair ^2a^, Valliamma Neelakandapilla Saritha ^2b^, Kunjuraman Sujathan ^2*^, Sreeja Sreeharshan^1*^

Cancer Research, Rajiv Gandhi Centre for Biotechnology,

Thiruvananthapuram, 695014,

Kerala, India.

1 & 1*- Cancer Research Program, Rajiv Gandhi Centre for Biotechnology,

Thiruvananthapuram, Kerala, India.

2^a^ - Cancer Epidemiology & Biostatistics, Regional Cancer Centre, Thiruvananthapuram, Kerala, India.

2^b^ and 2* - Cancer Research, Regional Cancer Centre, Thiruvananthapuram, Kerala, India.

Corresponding authors: *Sujathan K, *Sreeja Sreeharshan

For correspondence : Dr.S.Sreeja,

Scientist EII

Cancer Research Program,

Rajiv Gandhi Centre for Biotechnology,

Thiruvananthapuram, 695014,

Kerala, India

Tel: +91471 2529474

Or

K. Sujathan,

Additional Professor,

Regional Cancer Centre

Medical College Campus, Ulloor,

Thiruvananthapuram, Kerala 695011

###

### Email: ksujathan@gmail.com, [ssreeja@rgcb.res.in](mailto:ssreeja@rgcb.res.in)

**Supplementary Information-Figure legends.**

Supplementary Figure S1: **Ezrin expression and activation after E2 treatment.** (A) Western blot showing expression of phospho-ezrin in FTC-133 cells treated with E2 (10nM), ERβ agonist DPN (10nM), ERα agonist PPT (10nM) for 15 minutes. (B) Western blot analysis showing the expression of phospho-ezrin in FTC-133 cells pre-treated with cycloheximide (50µM) followed by E2 for 15 minutes. (C) Western blot showing expression of phospho-AKT in FTC-133 cells treated with E2 (10nM), MEK inhibitor PD 98059 (20µM), anti-estrogen ICI 182780 (1µM) and wortmannin (30nM) along with E2. P-AKT densitometry values were adjusted and normalized to the values of AKT. Phospho-ezrin densitometry values were adjusted and normalized to the values of wild type ezrin. Statistical significance was calculated using t-test * = p ≤ 0.05, ** = p ≤ 0.01 vs. control; # = p< 0.05, ## = p≤ 0.01 vs. E2 respectively.

Supplementary Figure S2: **Knock down of ezrin and inhibition of phosphorylation decreases follicular cancer cell migration and invasion**. (A) Western blot showing expression of phospho-ezrin in FTC-133 cells exposed to ROCK-2 siRNA. (B) Nearly confluent FTC-133 cells were wounded by using a 0.1-10µl tip and cells were exposed to E2 (10nM), PI3K inhibitor wortmannin (30nM) in the presence or absence of E2 (10nM). Images of denuded areas were taken at 0, 24 and 48hours respectively. (C) Representative micrographs showing FTC cells invaded after cells treated with E2 (10nM), wortmannin (30nM) in presence or absence of E2 (10nM).

Supplementary Figure S3: **Receiver operating curve.** ROC curve for histological association between (A) follicular thyroid carcinoma and follicular adenoma. (B) Follicular variant of papillary thyroid carcinoma and follicular adenoma. (C) Papillary thyroid carcinoma and follicular adenoma.

Supplementary Figure S4: (A) FTC-133 cells were exposed to E2 (10nM) for 6, 12, 24 and 48hours respectively. Representative image of western blot showing immunoreactive protein ERα in FTC-133 cells. (B) Western blot analysis showing expression of ezrin in ERα positive FTC-133 cells in response to E2 (10nM) treatment until 48 hours. (C) Corresponding loading control β-actin of A and B. (D) Western blot analysis showing expression of ezrin in ERα positive Nthy cells in response to E2 (10nM) treatment until 48 hours. (E) Corresponding loading control β-actin of D.

Supplementary Figure S5: (A) Western blot analysis showing expression of ezrin with increasing concentrations of E2 (10^-8^M, 10^-7^M, and 10^-6^ M). (B) Corresponding loading control β-actin (C) Western blot showing expression of ezrin in FTC cells treated with E2 (10nM), anti-estrogen ICI-182 780 (1µM) in presence or absence of E2 (10nM). (D) Corresponding loading control β-actin.

Supplementary Figure S6: (A) Western blot showing expression of ezrin in FTC-133 cells exposed to E2 (10nM), ER β agonist DPN (10nM) and ERα agonist PPT (10nM) for 24 hours. (B) Corresponding loading control β-actin (C) Western blot showixng expression of ezrin in ERα silenced FTC cells. (D) Expression of ERα in FTC cells exposed to ERα siRNA. (E) Corresponding loading control β-actin.

Supplementary Figure S7: (A) Western blot showing expression of phospho-ezrin in FTC-133 cells treated with increasing concentrations of E2 (10^-8^M, 10^-7^M, and 10-6 M) for 15 minutes. (B) Corresponding loading control β-actin. (C) FTC-133 cells were treated with E2 (10nM), ERβ agonist DPN (10nM), ER α agonist PPT (10nM) for 15 minutes. (D) Western blot showing total protein ezrin and loading control β- actin. (E) Western blot showing FTC cells treated with E2 for 5’, 10’, 15’, 30’ and 60’ respectively. (F) Corresponding loading control β-actin.

Supplementary Figure S8: (A) Western blot showing expression of phospho-ezrin in FTC cells exposed to Erα siRNA. (B) FTC-133 cells were treated with E2 (10nM), EBSA (10nM) and ICI (1µM) in the presence of E2 (10nM) for 15 minutes. (C) Western blot analysis showing the expression of phospho-ezrin in FTC-133 cells pre-treated with cycloheximide (50µM) followed by E2 for 15 minutes. (D) Western blot showing total protein ezrin and loading control β- actin.

Supplementary Figure S9: (A) Western blot showing expression of phospho-ezrin in FTC-133 cells exposed to E2 (10nM), MEK inhibitor PD 98059 (20µM) and PI3K inhibitor wortmannin (30nM) in presence or absence of E2. (B) Corresponding total protein ezrin. (C) Western blot showing corresponding loading control β- actin. (D) FTC-133 cells were treated with E2 (10nM), MEK inhibitor PD 98059 (20µM), antiestrogen ICI 182780 (1µM) and wortmannin (30nM) along with E2 and analysed the expression of phospho-AKT. (E) Total protein Akt. (F) Western blot showing loading control β- actin.

Supplementary Figure S10: (A) FTC- 133 cells were treated with E2 (10nM) and ROCK-2 inhibitor Y- 27632 (10µM) in presence or absence of E2. (B) and (C) Western blot showing total protein ezrin and loading control β- actin.

Supplementary Figure S11: (A) FTC-133 cells were exposed to ROCK-2 siRNA and checked the expression of phospho-ezrin. (B) Corresponding total protein ROCK-2. (C) Corresponding total protein ezrin. (D) Corresponding loading control β-actin.

Supplementary Figure 12: (A) Western blot analysis showing expression of ezrin in ezrin silenced FTC-133 cells. (B) western blot showing expression of 14-3-3 ζ in ezrin silenced cells. (C) Western blot showing expression of β-catenin in ezrin silenced cell. (D) Western blot showing expression of vimentin in ezrin silenced cells. (E) Western blot showing expression of snail in ezrin silenced cells. (F) Western blot showing expression of E- cadherin in ezrin silenced cells.

Supplementary Figure S13: (A) Loading control β-actin of ezrin silenced and scrambled control cells. (B) Western blot showing expression of N-cadherin in ezrin silenced cells. (C) Western blot showing expression of phospho-ezrin in FTC-133 and Nthy cells. (D) Corresponding loading control β-actin. (E) Corresponding total protein ezrin.

**Supplementary figure S1**


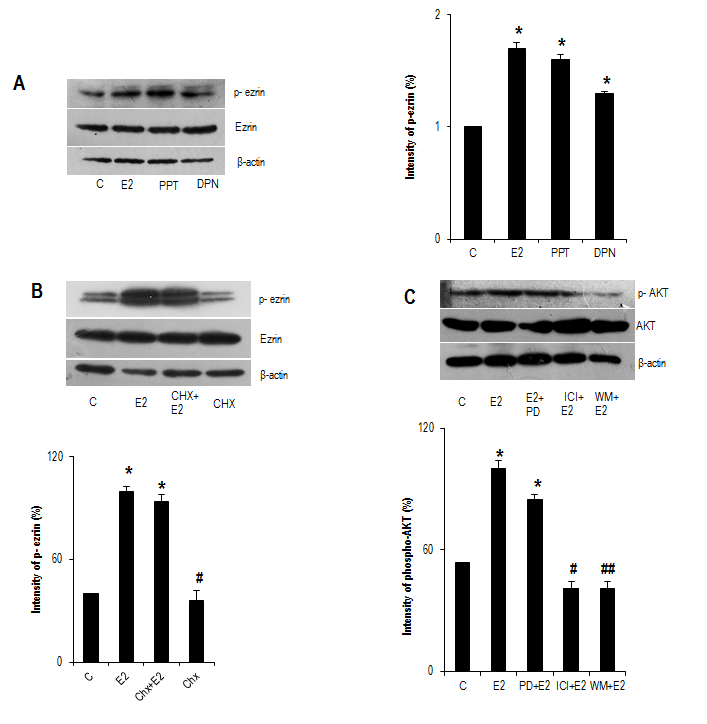


**Supplementary Figure S2**

**
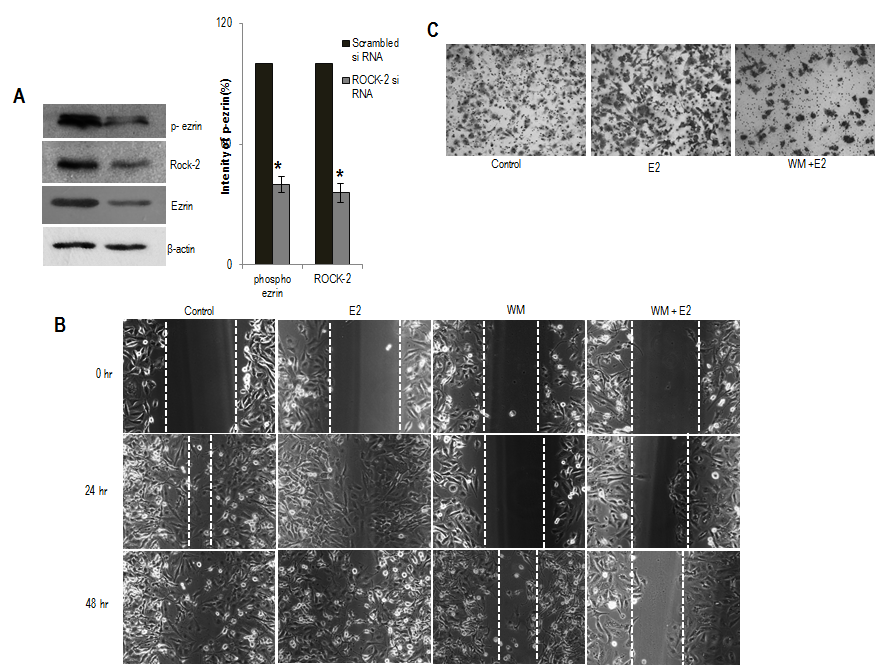
**

**
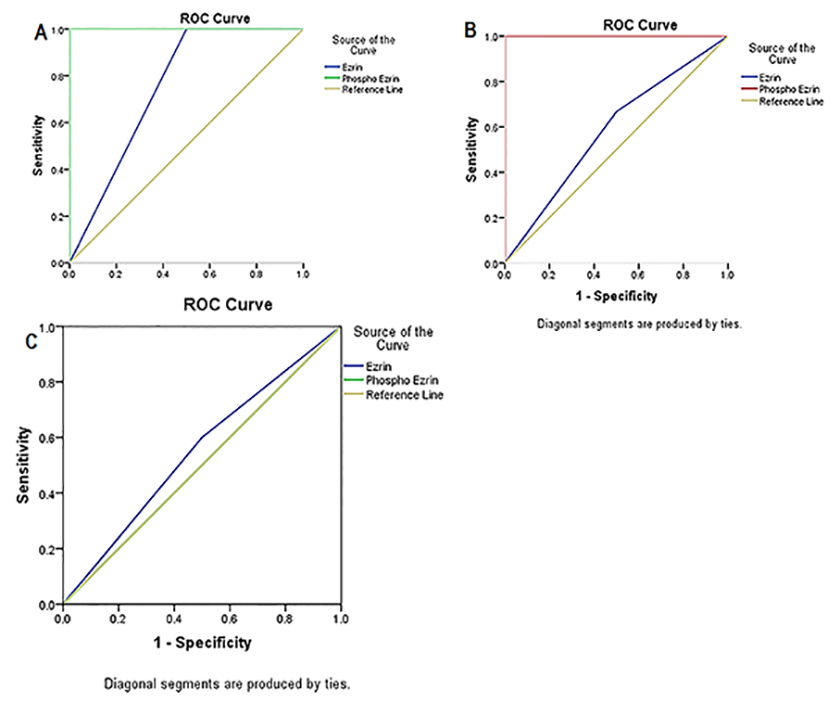
Supplementary Figure S3**

**Supplementary figure S4**

**
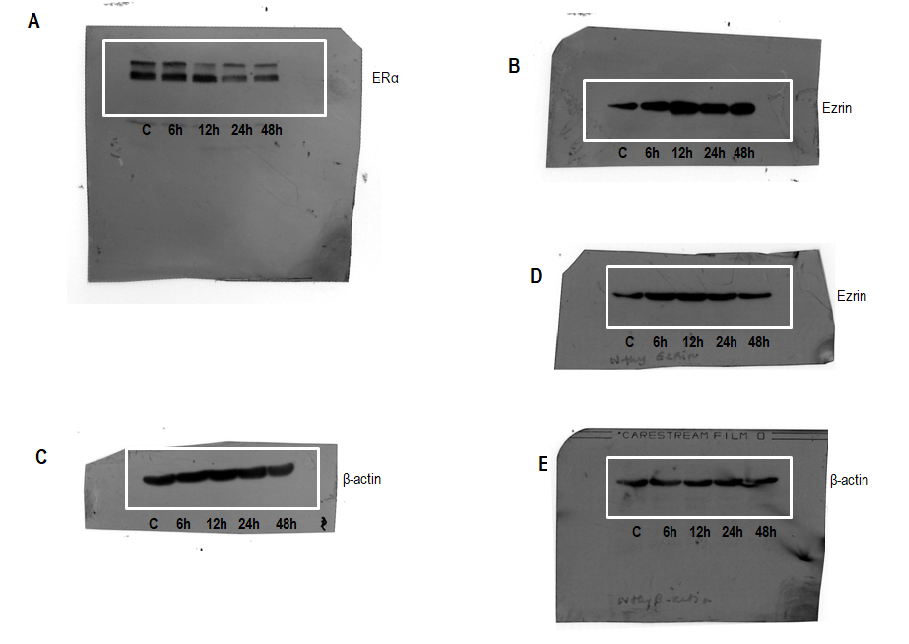
**

**
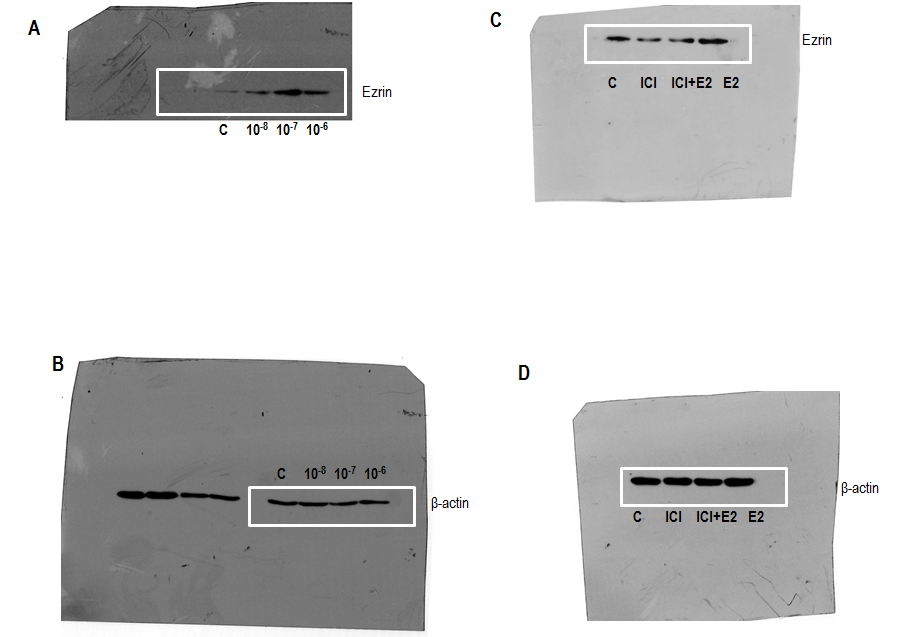
Supplementary figure S5:**

**Supplementary figure S6:**

**
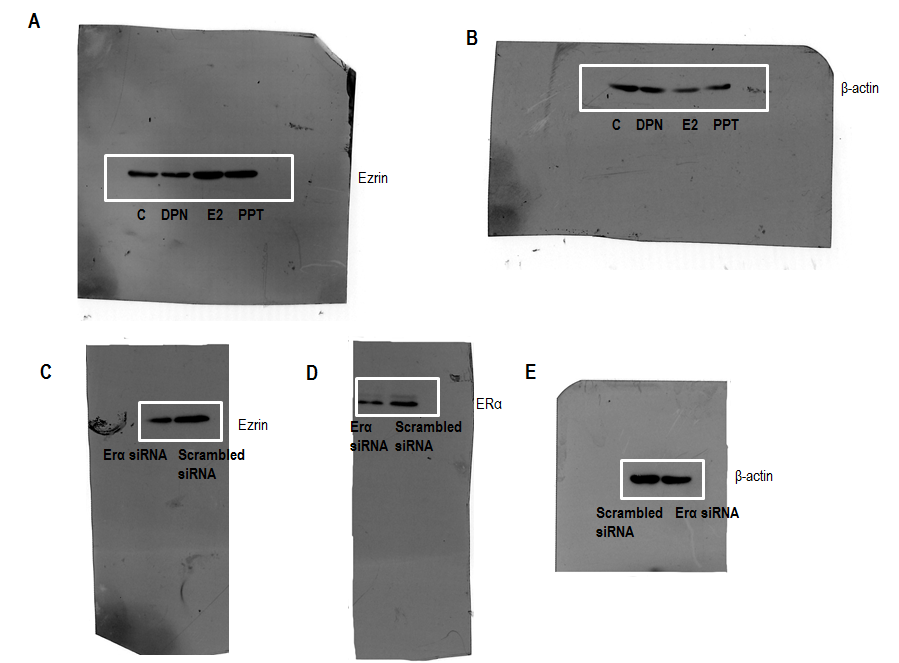
**

**
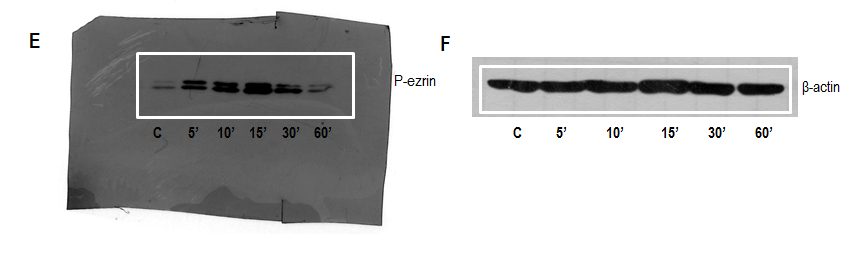

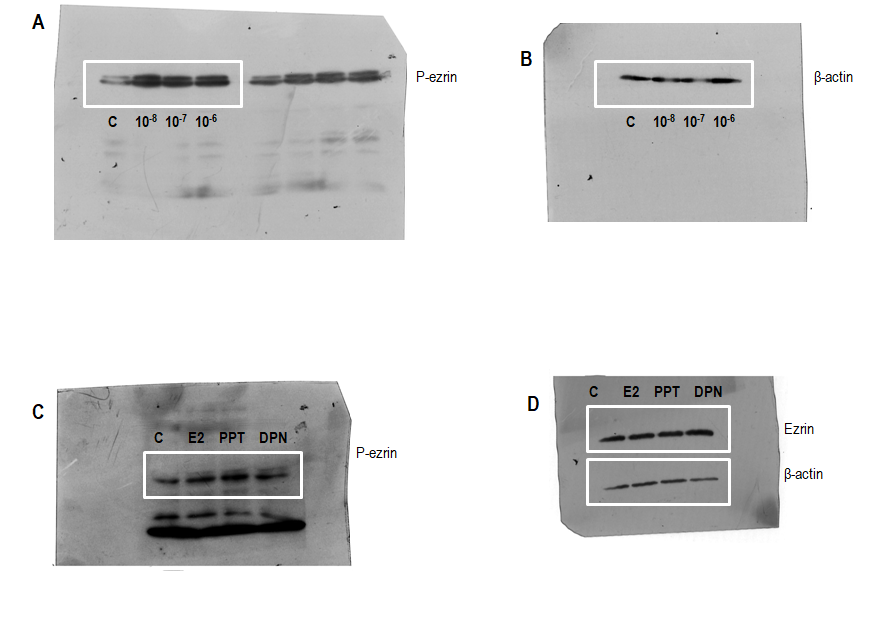
Supplementary figure S7:**

**Supplementary Figure S8**

**
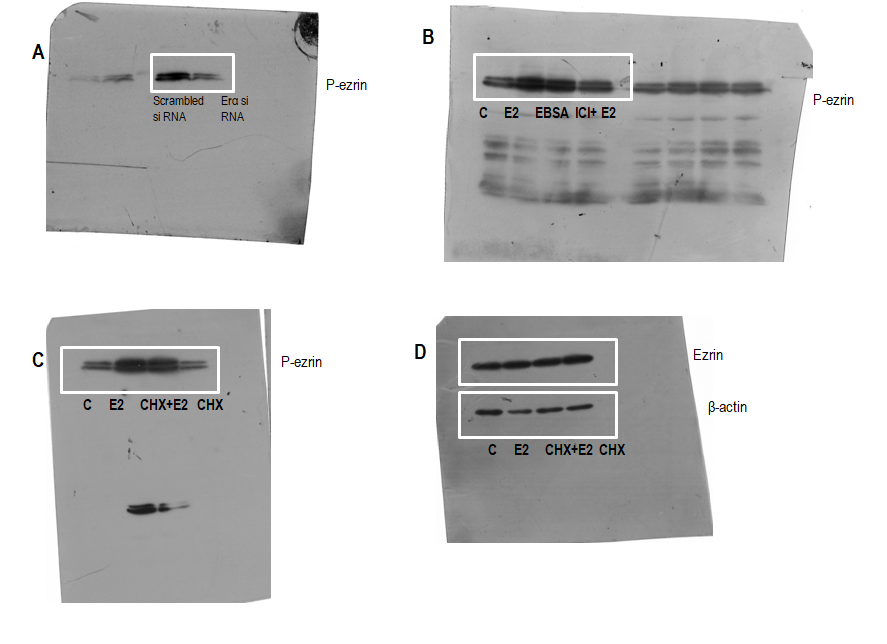
**

**Supplementary figure S9**

**
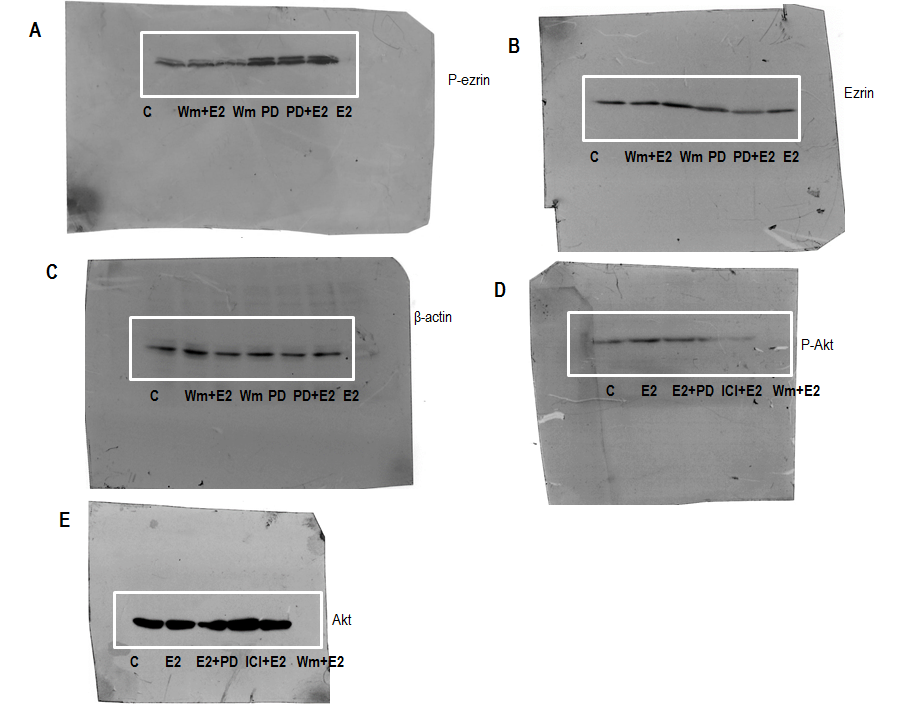
**

**F**


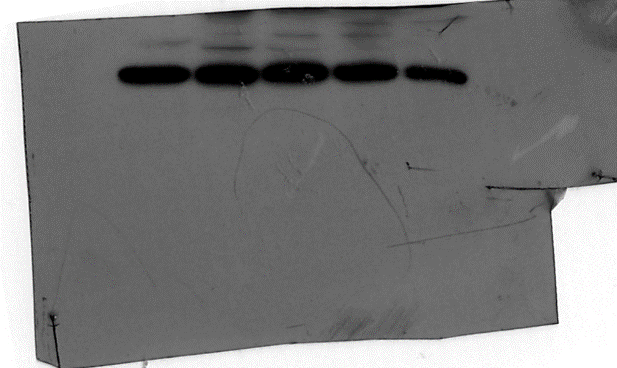


β-actin

**C E2 E2+PD ICI+E2 Wm+E2**

**Supplementary Figure S10**

**
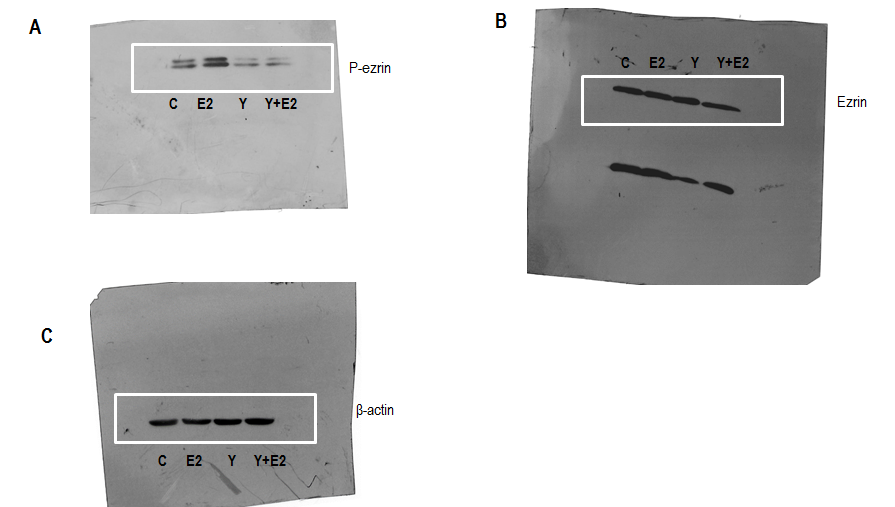
**

**
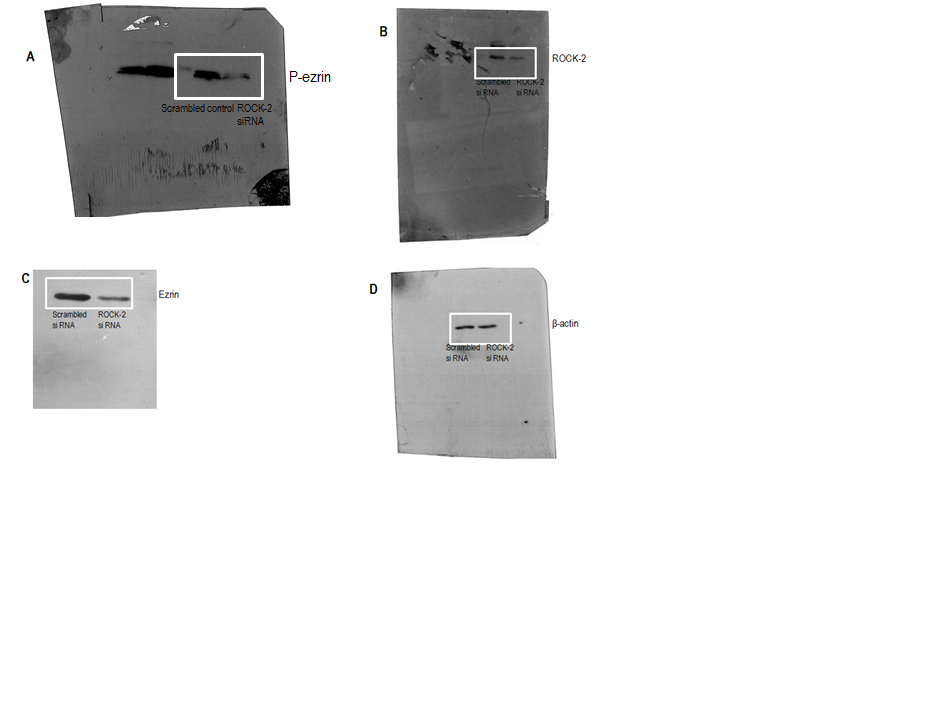
Supplementary figure S11**

**Supplementary Figure S12**

**
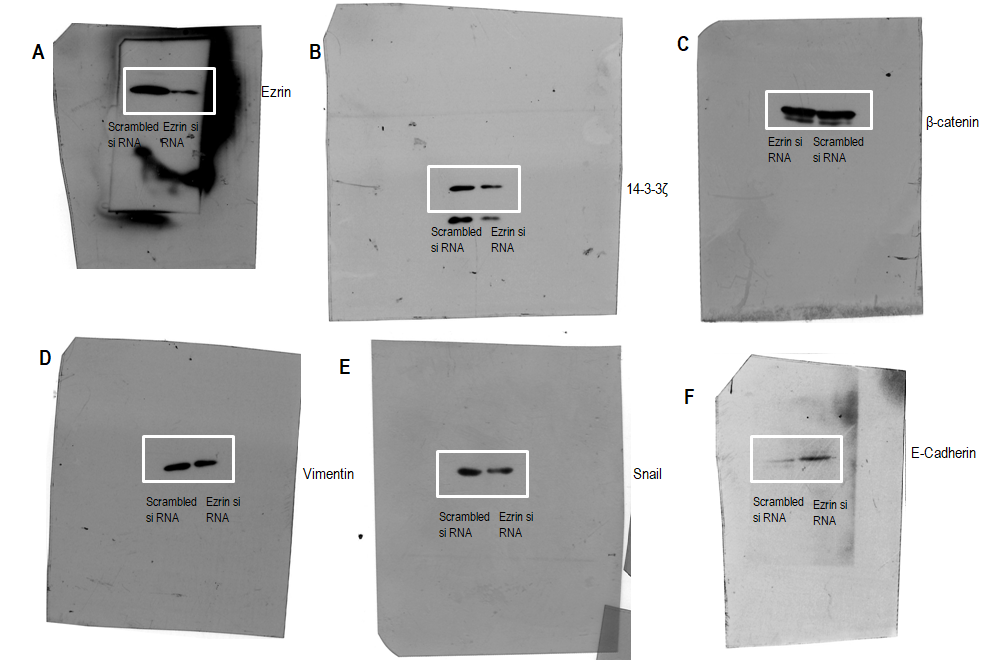
**

**
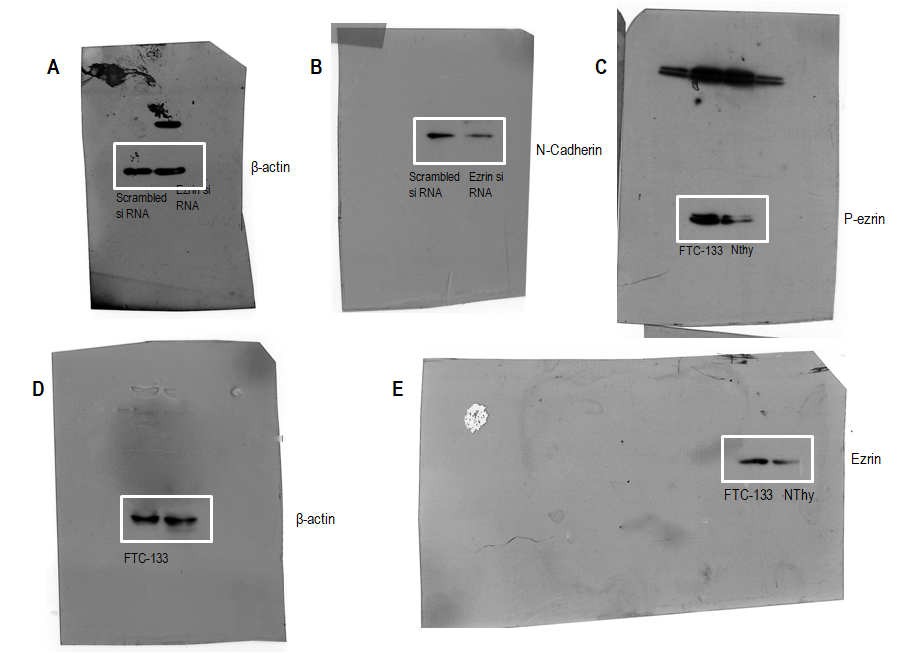
Supplementary Figure S13**

Nthy
